# Enhanced adsorption of ciprofloxacin from water using raw and phosphoric acid-modified cauliflower-leaves derived biochar: response surface optimization

**DOI:** 10.3389/fchem.2026.1852792

**Published:** 2026-07-23

**Authors:** Qazi saliq, Muhammad Rizwan, Sheeraz Ahmed Memon, Alin Ciobica, Vasile Burlui, Daniela Tomita, Hajira Haroon

**Affiliations:** 1 Department of Environmental Sciences, University of Haripur, Haripur, Khyber Pakhtunkhwa, Pakistan; 2 U.S.-Pakistan Center for Advanced Studies in Water, Mehran University of Engineering and Technology, Jamshoro, Pakistan; 3 Department of Environmental Engineering and Management, Mehran University of Engineering and Technology, Jamshoro, Pakistan; 4 Department of Biology, Faculty of Biology, Alexandru Ioan Cuza University of Iasi, Iasi, Romania; 5 CENEMED Platform for Interdisciplinary Research, “Grigore T. Popa” University of Medicine and Pharmacy, Iasi, Romania; 6 “Ioan Haulica” Institute, Apollonia University, Iasi, Romania; 7 Biomedical Research Group, “Olga Necrasov” Center, Romanian Academy, Iasi Branch, Teodor Codrescu, Iasi, Romania; 8 Academy of Romanian Scientists, Bucuresti, Romania

**Keywords:** adsorption, biochar, ciprofloxacin, isotherms, kinetics, response surface methodology

## Abstract

Ciprofloxacin (CIP), a widely prescribed fluoroquinolone antibiotic, has emerged as an environmental concern because of its persistence in aquatic systems, limited removal by conventional treatment processes, and role in the spread of antibiotic resistance. While biochar adsorption has gained attention for pharmaceutical removal, the use of cauliflower-leaf-derived biochar, particularly after chemical modification, has received limited investigation. Furthermore, studies integrating optimization of both biochar preparation and adsorption performance remain scarce. In the present study, raw cauliflower-leaf biochar (CLB10) and phosphoric-acid-modified biochar (PAM-CLB1.5) were evaluated for CIP removal from water using Response Surface Methodology based on a Box–Behnken experimental design. The optimized preparation conditions, i.e., pyrolysis time, temperature, and particle size for CLB10 were identified as 90 min, 475 °C, and 272.5 μm, respectively. Under optimized adsorption conditions, CLB10 achieved 78.43% CIP removal, whereas PAM-CLB1.5 exhibited substantially higher removal efficiency (97.54%), demonstrating the beneficial effect of phosphoric acid modification. Surface characterization showed that acid treatment enhanced pore development, increased specific surface area from 15.03 to 24.65 m^2^ g^-1^, and introduced phosphorus-containing functional groups that promoted stronger adsorbent–adsorbate interactions. CIP adsorption data were better described by the Langmuir isotherm model for both CLB10 and PAM-CLB1.5, suggesting predominant monolayer-type adsorption behavior under the investigated conditions. Kinetic data followed the pseudo-second-order model, indicating that adsorption was strongly influenced by surface-site-controlled interactions. Higher removal efficiency was attributed to the combined effects of pore filling, hydrogen bonding, electrostatic attraction, and π–π interactions. Regeneration experiments demonstrated satisfactory reusability, with the modified biochar retaining approximately 87% removal efficiency after five adsorption–desorption cycles. It was concluded that PAM-CLB1.5 could be used as an efficient adsorbent for the removal of CIP from water.

## 1 Introduction

Pharmaceuticals, particularly antibiotics, are increasingly recognized as emerging contaminants because of their extensive use and continuous release into aquatic systems (Al-Howri et al., 2025; Haider et al., 2024). Ciprofloxacin (CIP), a widely prescribed fluoroquinolone antibiotic, is frequently detected in aquatic environments because of high consumption, incomplete removal, and environmental persistence (Liu et al., 2024a). Environmental CIP contamination is concerning because it may induce ecotoxicological effects, alter microbial communities, and promote antibiotic resistance (Al-Buriahi et al., 2022). CIP residues may also influence soil microbial activity and facilitate the dissemination of antibiotic-resistance genes (Mishra et al., 2026; Pasket et al., 2022).

Several technologies, including biological treatment, adsorption, advanced oxidation, membrane filtration, and enzymatic degradation, have been investigated for CIP removal (Phoon et al., 2020). Biological treatment often exhibits limited efficiency because the antimicrobial nature of CIP suppresses microbial activity (Güney and Sponza, 2016). Although AOPs and membrane-based systems can achieve effective degradation or separation, their application may be constrained by operational cost, energy requirements, and process complexity (Ahmed et al., 2022; Malakootian et al., 2020). Consequently, adsorption is considered an attractive alternative because of its simplicity, cost-effectiveness, and limited generation of toxic by-products (Ajala et al., 2023; Xiaoxiao and Jingchan, 2024).

Various adsorbents, including activated carbon, clays, carbon nanotubes, and biochar, have been explored for CIP removal (Haider et al., 2025; Krasucka et al., 2021; Malakootian et al., 2021; Sajid et al., 2022). Biochar is widely explored because of its higher porosity, adaptable surface chemistry, and sustainable production from biomass residues (Qiu et al., 2022). Its porous structure and surface functionalities support multiple adsorbent–adsorbate interactions relevant to CIP uptake (Igwegbe et al., 2021; Qiu et al., 2022). However, adsorption efficiency strongly depends on feedstock selection, pyrolysis conditions, and post-production surface modification (Li et al., 2020; Qiu et al., 2022).

Chemical modification is commonly applied to improve biochar adsorption performance by altering pore structure and surface functionality (Haider et al., 2025). For example, chemically modified bamboo biochar exhibited improved CIP removal relative to unmodified biochar (Wakejo et al., 2022), while H_3_PO_4_-modified Sidr biochar showed enhanced adsorption performance attributable to improved porosity and phosphorus-containing functionalities (Iqbal et al., 2022). Similarly, rabbit manure biochar demonstrated effective CIP adsorption, confirming the importance of feedstock characteristics and surface chemistry in determining adsorption behavior (Huang et al., 2020).

Cauliflower leaves represent an abundant agricultural residue with potential for sustainable biochar production. Previous studies demonstrated that cauliflower-leaf-based materials can function as low-cost adsorbents for contaminant removal from aqueous systems (Ansari et al., 2016). Biochar derived from cauliflower leaves has also shown favorable adsorption characteristics, including suitable porosity and oxygen-containing functional groups contributing to metal uptake. Furthermore, phosphoric acid modification has been reported to significantly improve biochar physicochemical properties by increasing porosity, surface functionality, and phosphorus-containing active sites that facilitate adsorption through ion exchange, complexation, and electrostatic interactions (Zeng et al., 2022).

Despite increasing interest in biochar-based removal of pharmaceutical contaminants, information regarding cauliflower-leaf-derived biochar for CIP remediation remains limited, particularly in chemically modified form. Moreover, optimization of biochar preparation and adsorption performance is commonly performed separately rather than through an integrated framework.

Therefore, the present study investigated CIP removal from water using raw cauliflower-leaf biochar (CLB10) and phosphoric-acid-modified cauliflower-leaf biochar (PAM-CLB1.5). Response Surface Methodology (RSM), Box–Behnken Design (BBD) was employed to optimize both biochar production parameters (pyrolysis time, temperature, and biomass particle size) and adsorption conditions affecting CIP removal.

There are two main aspects in the novelty of the current research. First, to the best of our knowledge, no study has been carried out to date comparing raw (unmodified) and modified biochar prepared from cauliflower leaves for ciprofloxacin adsorption using Response Surface Methodology (RSM). Second, the present study systematically applies RSM to optimize both biochar production conditions and batch adsorption parameters, providing a comprehensive evaluation of adsorbent preparation and performance. The prepared adsorbents were characterized using FTIR, SEM, EDX, and BET analyses, while adsorption behavior and mechanisms were evaluated using kinetic, isotherm, and regeneration studies.

## 2 Materials and methods

### 2.1 Chemical and materials

Cauliflower leaves were collected from Haripur District, Khyber Pakhtunkhwa, Pakistan, during the harvesting season. All chemicals, including hydrochloric acid (HCl), nitric acid (HNO_3_), phosphoric acid (H_3_PO_4_), oxalic acid (C_2_H_2_O_4_), sodium hydroxide (NaOH), potassium hydroxide (KOH), ammonium hydroxide (NH_4_OH), sodium bicarbonate (NaHCO_3_), Zinc chloride (ZnCl_2_), calcium chloride (CaCl_2_), potassium chloride (KCl), and methanol used in this study were analytical grade, purchased from Sigma Aldrich Co. (United States of America). Ciprofloxacin hydrochloride (CIP) analytical grade with purity >99.6% was purchased from Sigma Aldrich Co. (United States of America). A 1000 mg L^-1^ stock solution was prepared by dissolving 1000 mg CIP in 1000 mL double-deionized water. This stock solution was further used in the preparation of subsequent solutions of desired concentration for various batch design experiments.

### 2.2 Biochar preparation using RSM

Collected biomass was thoroughly washed with deionized water to remove adhering impurities. To ensure complete moisture removal, the biomass was further oven-dried at 60 °C for 12 h. The dried biomass was milled and sieved into particle-size fractions ranging from 40 to 420 μm using a set of variable pore size sieves, i.e., 40, 60, 80, 100, 125, 272, and 420 mesh. Finally, the biomass was transferred to air-tight bags for storage before biochar production. Biochar was produced by slow pyrolysis in a laboratory furnace under a continuous nitrogen flow rate of 100 mL min^-1^ to ensure an inert atmosphere during pyrolysis. Samples were heated at 10 °C min^-1^ under controlled pyrolysis conditions. (Lehmann and Joseph, 2015). RSM was used to optimize the impacts of three independent factors during biochar preparation, including pyrolysis time/holding time (60–120 min), pyrolysis temperature (300 °C–650 °C), and biomass particle size (125–450 µm) on the percentage removal of CIP. In the present research, the experiments were conducted using BBD. The experimental design generated 17 runs, consisting of 12 factorial (edge) points and five replicated center points. The center-point replicates were incorporated to estimate pure experimental error, assess reproducibility, and evaluate the adequacy of the quadratic regression model. Low and high factor levels were selected from literature reports. The BBD generated three coded levels (−1, 0, +1) for each variable, as presented in Table 1, wherein the levels for the BBD were shown together with the coded values that corresponded to them. Seventeen biochar samples were generated according to the BBD matrix (Table 5; Supplementary Table A1).

**TABLE 1:** The independent variables and their levels in the BBD experimental design for biochar preparation.

S. No	Variables	−1	0	1
1	Time (min)	60	90	120
2	Temperature (°C)	300	475	650
3	Particle Size (µm)	125	272.5	420

### 2.3 Biochar batch screening experiment

The CIP removal potential of the prepared seventeen biochars (Supplementary Table A1) was explored in the batch experiments. The experimental conditions of adsorbent dose (50 mg), temperature (30 °C), solution pH (7), CIP concentration (50 mgL^-1^), particle size (420 μm), and agitation (220 rpm) were kept constant for all the prepared biochar adsorbents. The CIP adsorption potential of all the biochar was screened for 24 h in the shaking incubator, having a fuzzy control system (WIS-20, Korea). One biochar, on the basis of the highest removal efficiency, i.e., CLB10 (67.06% CIP removal), as mentioned in Supplementary Table A1, was chosen for further batch experimentation. The biochar with the lowest removal efficiency, i.e., CLB15 (47.78% CIP removal) as shown in Supplementary Table A1 was selected for chemical modification.

### 2.4 Chemical modification of biochar

The biochar showing the lowest adsorption performance (CLB15) was selected for chemical modification (47.78% CIP removal) as mentioned in Supplementary Table A1. The reason for selecting the biochar with lower removal efficiency for chemical modification was to evaluate whether surface functionalization could significantly enhance its adsorption performance.

The chemicals used for modification are mentioned in Table 2. Fresh solutions (0.5, 1.5, and 2.5 M) were prepared using analytical-grade reagents and deionized water.

**TABLE 2:** Chemicals used for the modification of biochar.

S. No	Strength/Type	Acid	Base	Salt
1	Strong	Nitric acid (HNO_3_)	Potassium hydroxide (KOH)	Zinc chloride (ZnCl_2_)
2	Moderate	Phosphoric acid (H_3_PO_4_)	Ammonium hydroxide (NH_4_OH)	Calcium chloride (CaCl_2_)
3	Weak	Oxalic acid (C_2_H_2_O_4_)	Sodium bicarbonate (NaHCO_3_)	Potassium chloride (KCl)

A 1.5 g biochar was mixed with 50 mL of the respective solution in a 100 mL beaker, sealed with parafilm, and agitated on a shaker at 220 rpm for 24 h at 30 °C. After treatment, the mixture was filtered through a 0.2 μm cellulose nitrate membrane filter, and the filtrate was repeatedly washed with deionized water to achieve neutral pH. Modified biochars were oven-dried, cooled, and stored in labeled airtight glass vials.

### 2.5 Screening of modified biochar

The CIP removal potential of the 27 modified biochars, as mentioned in Supplementary Table A2, was explored in the batch experiments. The screening experimental conditions already discussed in Section 2.3 were kept constant for all the modified biochars. The CIP adsorption potential of all the biochar was screened for 24 h in the shaking incubator, having a fuzzy control system (WIS-20, Korea), under the set conditions of temperature (30 °C) and shaking speed (220 rpm). The biochar with the highest removal efficiency (82.71% CIP removal), abbreviated as PAM-CLB1.5 in Supplementary Table A2, was selected for further batch studies.

### 2.6 Adsorbent characterization

FTIR analysis of the selected biochar was performed to identify the surface functional groups involved in the adsorption process. The dried adsorbent samples (before and after adsorption) were mixed with KBr, pressed into pellets, and subsequently analyzed to obtain the FTIR spectra. SEM analysis was conducted to observe the surface morphology of the adsorbents. For this purpose, the samples were mounted on brass stubs using double-sided adhesive tape, and SEM images were captured at different magnifications (×300 to 1600x) using a JSM-5910 scanning electron microscope. The micrographs were obtained at an accelerating voltage of 20 kV with a secondary electron detector, while maintaining a working distance of 17 mm. The adsorption of CIP onto the adsorbent surface was further supported by EDX analysis. In addition, the specific surface area of the adsorbent was determined by Brunauer–Emmett–Teller (BET) analysis using a Gemini VII 2390 (V1.03) instrument at 77 K under a N_2_ atmosphere. The pore volume was calculated from the volume of nitrogen adsorbed at a relative pressure of p/p° = 0.99, and the pore size was estimated using the BET relation 4V/A.

#### 2.6.1 Determination of pH_p_zc

The point of zero charge (pH_p_zc) of both CLB10 and PAM-CLB1.5 was determined using the pH drift method in which 0.01 g of adsorbent was added to a series of 50 mL aqueous solutions of 0.1 M NaCl with their initial pH values adjusted across a range, from pH 2 to pH 12, using 0.1 M HCl and 0.1 M NaOH. The solutions were then shaken at 200 rpm for 24 h at room temperature. The final pH of each solution was measured. The difference between the initial and final pH (ΔpH) was plotted against the initial pH, and the pH_p_zc corresponds to the point where this curve intersects the pH axis (where ΔpH = 0). At this specific pH, the surface of the biochar exhibits zero net charge (Guilhen et al., 2019).

### 2.7 Batch experiments

Batch adsorption experiments for CIP removal using CLB10 and PAM-CLB1.5 were conducted by varying four key factors: contact time (20–120 min), adsorbent dose (10–50 mg), solution pH (2–12), and initial CIP concentration (10–50 mg/L), as summarized in Table 3. All adsorption experiments were conducted at a constant temperature of approximately 30 °C to maintain controlled experimental conditions. Temperature was fixed to isolate the effects of the selected operational variables and to simulate practical, energy-efficient water treatment conditions without external heating. Each experiment involved mixing a fixed volume of CIP solution with a defined biochar dose under controlled conditions, followed by filtration and concentration analysis via UV-Vis spectrophotometry (Shimadzu UV-1800).

**TABLE 3:** The independent variables and their levels in the BBD experimental design for batch studies.

S. No	Variables	−1	0	1
1	Time (min)	20	70	120
2	Adsorbent dose (mg)	10	30	50
3	Solution pH (−)	2	7	12
4	CIP concentration (mg L^-1)^	10	30	50

The removal efficiency (%) was calculated by using Equation 1

$$\text{Removal efficiency}=\frac{\left(C_{0-}C_{e}\right)}{C_{0}}\times 100$$ (1)

To optimize these process variables and their interactions efficiently, BBD under RSM was employed. BBD is a statistical design that requires fewer experimental runs than full-factorial designs while allowing the modeling of quadratic relationships among variables. The response, Y (adsorption capacity or removal efficiency), was modeled by a second-order polynomial equation:

$$y=\beta_{0}+\sum_{i=1}^{k}\beta_{i}x_{i}+\sum_{i=1}^{k}\beta_{ii}x_{i}^{2}+\sum_{i=1}^{k-1}\sum_{j=2}^{k}\beta_{ij}x_{i}x_{j}+\varepsilon$$ (2)

where β_0_ is the intercept, β_i_ is the linear coefficient, β_ii_ is the quadratic coefficient, and β_ij_ is the interaction coefficient for independent variables X_i_ and X_j_ in Equation 2. This model enabled the evaluation of the individual and interactive effects of the four factors on CIP adsorption, thereby enabling precise prediction of optimal conditions (Montgomery, 2017). Batch adsorption optimization was conducted using a four-factor, three-level Box–Behnken Design (BBD) considering contact time (A), solution pH (B), initial CIP concentration (C), and adsorbent dose (D) as independent variables. The experimental matrix consisted of 30 runs, including 24 factorial (edge) points and six replicated center points. The center-point experiments were performed at 70 min contact time, pH 7, 30 mg L^-1^ initial CIP concentration, and 30 mg adsorbent dose. These replicated center points were included to estimate pure experimental error, validate model predictability, and assess adequacy of the quadratic response surface model.

### 2.8 Ciprofloxacin analysis

Ciprofloxacin concentration was analyzed using a double-beam UV-Vis spectrophotometer (Shimadzu UV-1800) at 274.8 nm.

### 2.9 Isotherm

Adsorption isotherm studies were conducted to evaluate the equilibrium adsorption capacity of biochar adsorbents at initial CIP concentrations of 10, 30, 50, 70, and 90 mg L^-1^. To maintain consistency, the experiments were performed under fixed conditions, including an adsorbent dose of 50 mg, particle size of 420 μm, temperature of 30 °C, solution pH of 7, and agitation speed of 220 rpm. The experimental equilibrium data were analyzed using two widely applied isotherm models: Langmuir and Freundlich. The Langmuir isotherm, describing monolayer adsorption on a homogeneous surface, was linearized in Equation 3 as

$$\frac{C_{e}}{q_{e}}=\frac{1}{Q_{m}b}+\frac{C_{e}}{Q_{m}}$$ (3)

In Equation 3, $C_{e}$ is the equilibrium concentration (mg L^-1^), $q_{e}$ is the adsorbed amount at equilibrium (mg g^-1^), $Q_{m}$ represents maximum monolayer adsorption capacity, and b denotes the Langmuir constant related to adsorption affinity. The Freundlich isotherm, suitable for heterogeneous multilayer adsorption, was expressed in a nonlinear form in Equation 4 as

$$\log q_{e}=\log K_{F}+\frac{1}{n}\log C_{e}$$ (4)

where $K_{F}$ indicates the adsorption capacity and 1/n describes adsorption intensity or surface heterogeneity. These models helped in interpreting the adsorption mechanism and capacity of biochar towards CIP, facilitating comparative analysis of the adsorbents and guiding adsorption process design (Al-Ghouti and Da’ana, 2020).

### 2.10 Kinetics

Kinetic studies were performed to evaluate the adsorption of CIP onto biochar using four different initial concentrations (30, 50, 70, and 90 mg L^-1^). Experiments were conducted at pH 7, with a biochar dose of 50 mg and particle size of 420 μm, maintained at 30 °C. CIP removal was monitored over various contact times ranging from 5 to 120 min. The adsorption data were analyzed using pseudo–first-order and pseudo–second-order kinetic models (Largitte and Pasquier, 2016).

The pseudo–first-order model is expressed in Equation 5 as

$$\log\left(q_{e}-q_{t}\right)=\log q_{e}-\frac{k_{1}}{2.303}t$$ (5)

where $q_{t}$ and $q_{e}$ are the adsorbed amounts at time t and equilibrium (mg/g), respectively, and $k_{1}$ is the rate constant.

The pseudo–second-order model is given in Equation 6

$$\frac{t}{q_{t}}=\frac{1}{k_{2}q_{e}^{2}}+\frac{t}{q_{e}}$$ (6)

In Equation 6 value of $k_{2}$ (g mg ^-1^ min ^-1^) is the rate constant for the pseudo-second-order kinetic model. The adsorption capacity, denoted as $q_{e}$ (mg g^-1^) represents the maximum amount of CIP adsorbed onto the biochars at equilibrium. The amount of CIP adsorbed at any time t (min) is represented as $q_{t}$ . Additionally, h (mg g^-1^ hr^-1^) corresponds to the initial adsorption rate, which is calculated using the relation $h=k_{2}q_{e}^{2}$ . These parameters $q_{e}$ , $k_{2}$ , and h are derived from the slope and intercept of the linear plot of $t/q_{t}$ versus t . This method allows for a comprehensive understanding of the adsorption dynamics, indicating how quickly and efficiently CIP is removed over time, with the kinetic studies typically confirming that the process adheres to pseudo-second-order kinetics, often associated with chemisorption mechanisms (Hamad et al., 2025).

### 2.11 Adsorbent regeneration

The regeneration of both biochars was examined for five consecutive adsorption-desorption cycles at an optimal operating conditions (pH = 7; CIP concentration = 10 mg L^-1^; Adsorbent dose = 50 mg L^-1^, time = 90 min, shaking speed = 220 rpm, and temperature = 30 °C) obtained from batch experiments. CIP desorption optimization experiments were conducted using 0.3M NaOH, 0.3M HCl, methanol, and 3% NaOH + methanol to get the best desorbing solution for the CIP.

The percentage (%) desorption was calculated by using the following Equation 7.

$$\%\text{ desorption}=\left(\frac{\text{amount}\;\text{of}\;\text{CIP}\;\text{desorbed}}{\text{amount}\;\text{of}\;\text{CIP}\;\text{adsorbed}}\right)\times 100$$ (7)

The CLB10 and PAM-CLB1.5 reusability test was then conducted using the best desorbing solution. After each cycle, CLB10 and PAM-CLB1.5 were thoroughly washed with ultrapure water, filtered, and dried in an oven, and then reused in the next adsorption cycle (Abu Rumman et al., 2021).

## 3 Results and discussions

### 3.1 Biochar characterization

#### 3.1.1 SEM

**FIGURE 1:**
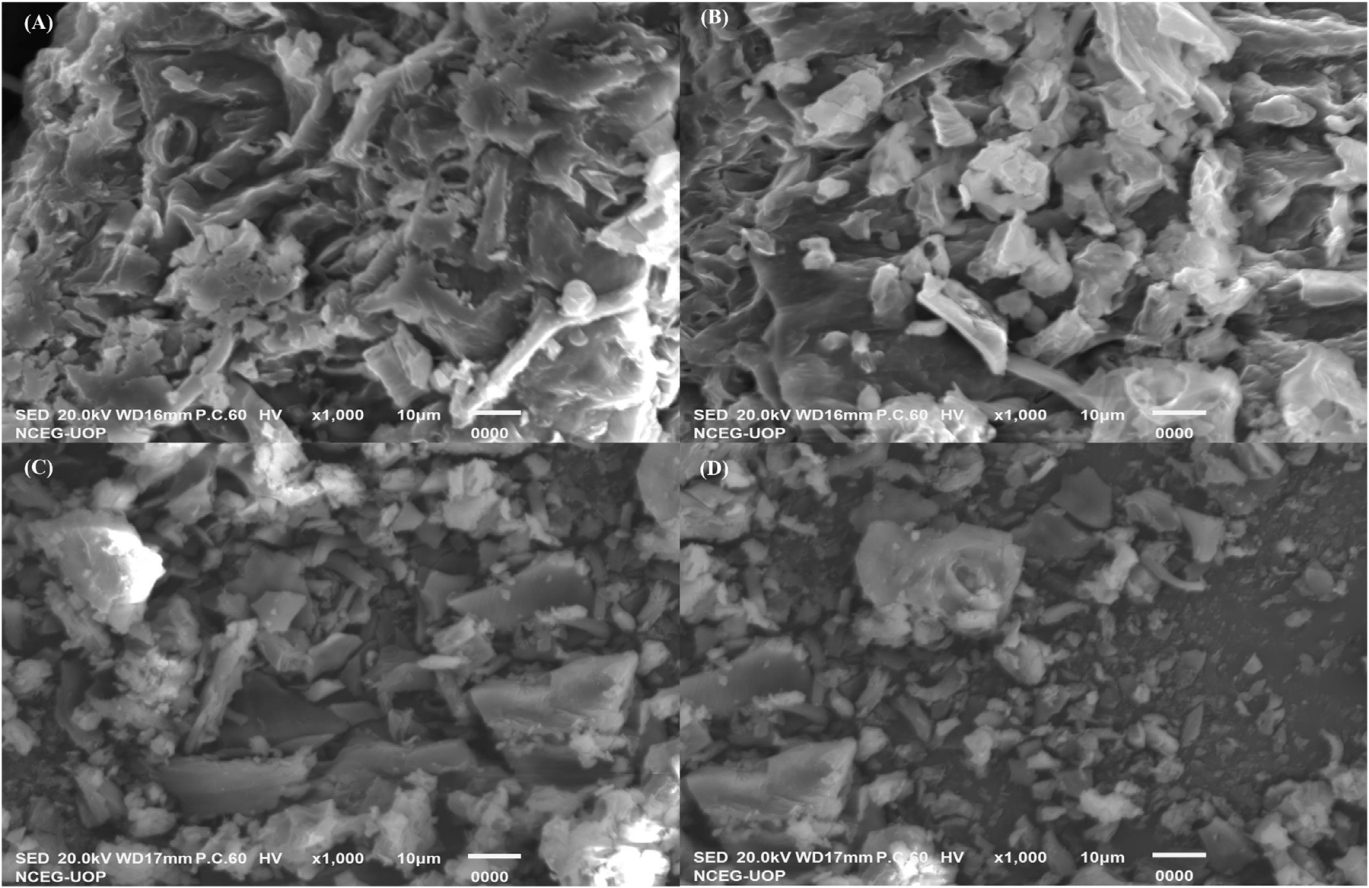
SEM Analysis of **(A)** CLB10 before adsorption **(B)** after adsorption, **(C)** PAM-CLB1.5 before adsorption, and **(D)** after adsorption.

SEM analysis was performed to compare morphological changes in CLB10 and PAM-CLB1.5 before and after CIP adsorption, as shown in Figures 1A–D. SEM analysis revealed clear morphological differences before and after adsorption for both biochars. Figure 1A, representing unmodified biochar (CLB10), exhibited a relatively rough and porous surface with visible micro- and mesoporous features generated during oxygen-limited pyrolysis. These features indicate the availability of accessible adsorption sites relevant to pollutant uptake (Zhao et al., 2018).

After CIP adsorption, Figure 1B shows that the CLB10 surface became comparatively smoother with partial pore coverage and localized surface accumulations. Reduced pore visibility and localized surface deposits indicate adsorption-induced surface occupation and pore filling. Figure 1C, corresponding to phosphoric-acid-modified biochar (PAM-CLB1.5). PAM-CLB1.5 exhibited greater porosity, stronger surface heterogeneity, and improved textural development relative to CLB10. These characteristics are consistent with the role of H_3_PO_4_ activation in promoting pore development and increasing accessible surface area, which is further supported by the BET results presented in Section 3.1.4 and Table 4. H_3_PO_4_ modification may additionally introduce oxygen- and phosphorus-containing functionalities that enhance adsorption affinity (Du et al., 2025), which is further supported by FTIR findings in Section 3.1.2. Following CIP adsorption, Figure 1D reveals increased surface coverage, particle aggregation, and reduced visible porosity in PAM-CLB1.5 compared with its pre-adsorption state. These observations indicate occupation of adsorption sites and interaction between CIP molecules and the modified adsorbent surface. The more pronounced morphological changes observed for PAM-CLB1.5 relative to CLB10 are consistent with its higher adsorption performance. Collectively, the SEM results suggest that phosphoric acid modification enhanced pore accessibility and reactive surface characteristics, thereby facilitating stronger adsorbent–adsorbate interactions involving surface coverage, pore filling, hydrogen bonding, electrostatic attraction, and π–π interactions rather than serving as standalone evidence of multilayer adsorption (Li et al., 2018).

**TABLE 4:** BET analysis of CLB10 and PAM-CLB1.5.

S. No	Biochar	BET surface area	Pore volume	Pore size
1	CLB10	15.032 m^2^/g	0.064 cm^3^/g	4.593 nm
2	PAM-CLB1.5	24.654m^2^/g	0.154 cm^3^/g	5.755 nm

#### 3.1.2 FTIR

**FIGURE 2:**
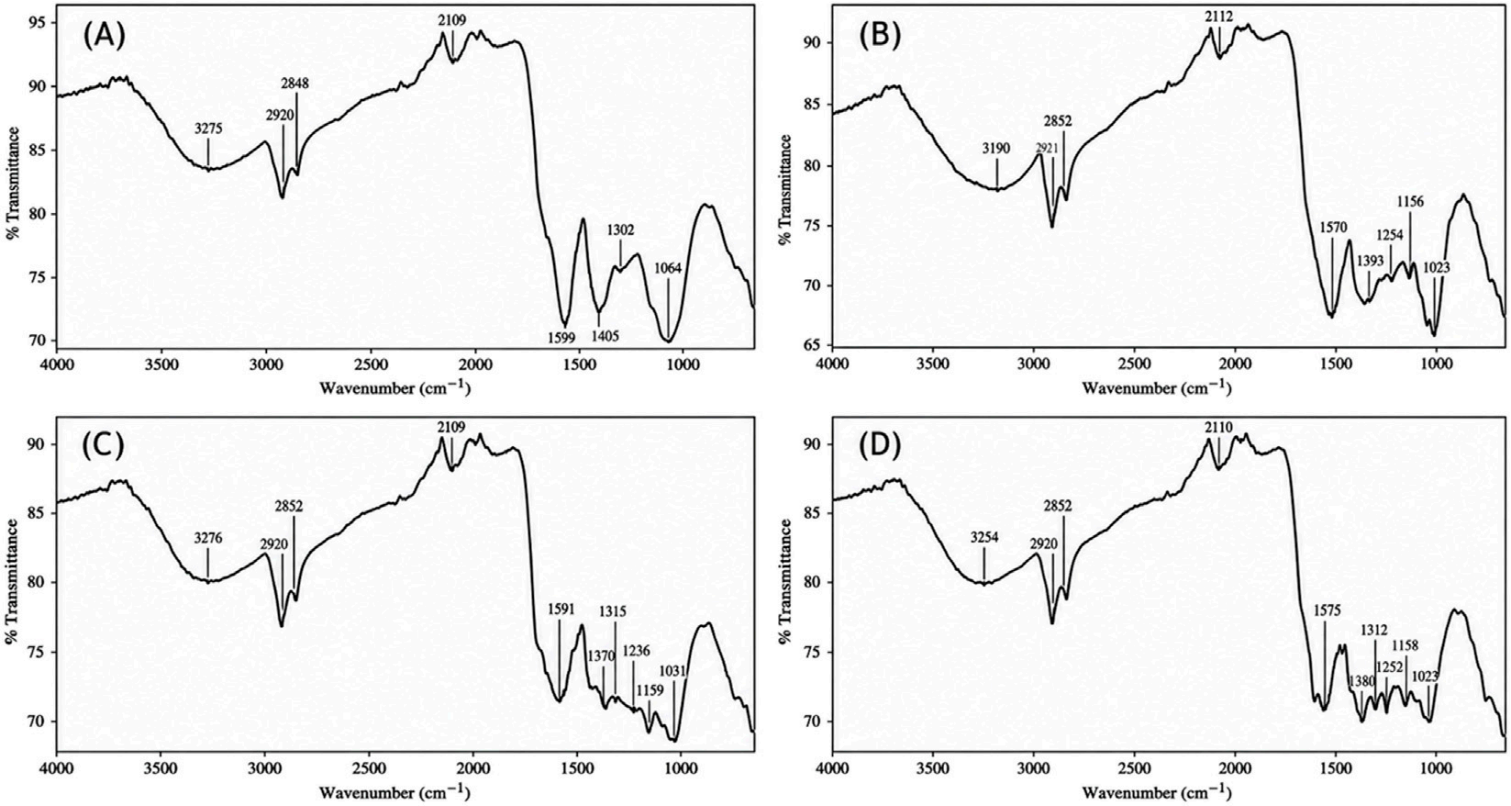
FTIR spectra of CLB10 before adsorption **(A)**, after adsorption **(B)** PAM-CLB1.5 before adsorption** (C)** and after adsorption **(D)**.

FTIR analysis was performed to compare surface functional groups on CLB10 and PAM-CLB1.5 before and after CIP adsorption (Figure 2). For pristine CLB10 (Figure 2A), the broad band at 3275 cm^-1^ corresponds to–OH stretching of hydroxyl/phenolic groups, while peaks at 2920 and 2848 cm^-1^ are assigned to aliphatic C–H stretching. The band near 1569 cm^-1^ indicates aromatic C=C structures, whereas peaks at 1405, 1302, and 1064 cm^-1^ correspond to–COO^-^ and C–O functionalities, confirming the presence of oxygen-containing surface groups relevant to adsorption (Kim et al., 2011; Shao et al., 2024).

Compared with CLB10, PAM-CLB1.5 (Figure 2C) exhibited distinct spectral changes after phosphoric-acid treatment, particularly the appearance/intensification of peaks at 1591, 1376, 1315, 1236, 1159, and 1031 cm^-1^, attributed to P=O/–PO_3_, P–O–C, and P–O groups. These changes confirm successful phosphate functionalization and demonstrate that H_3_PO_4_ modification enriched the biochar surface with oxygen- and phosphorus-containing acidic groups, consistent with the observed decrease in pH_p_zc and improved adsorption uptake.

After CIP adsorption, both biochars showed measurable peak shifts, but the changes were more pronounced for PAM-CLB1.5. For CLB10 (Figure 2B), shifts from 3275→3190, 1405→1393, 1302→1254/1156, and 1604→1023 cm^-1^ suggest involvement of–OH, –COO^-^, and C–O groups in adsorption. In PAM-CLB1.5 (Figure 2D), shifts at 3275→3254, 1591→1575, 1376→1380, 1315→1313, and 1236→1252 cm^-1^ indicate participation of–OH, aromatic C=C, P=O/–PO_3_, P–O–C, and P–O functionalities. The stronger spectral response of PAM-CLB1.5 suggests that phosphoric-acid modification generated additional chemically active sites, enhancing CIP uptake through hydrogen bonding, electrostatic interactions, Lewis’s acid–base interactions, and π–π interactions, rather than through textural improvement alone (Haider et al., 2026).

#### 3.1.3 EDX

**FIGURE 3:**
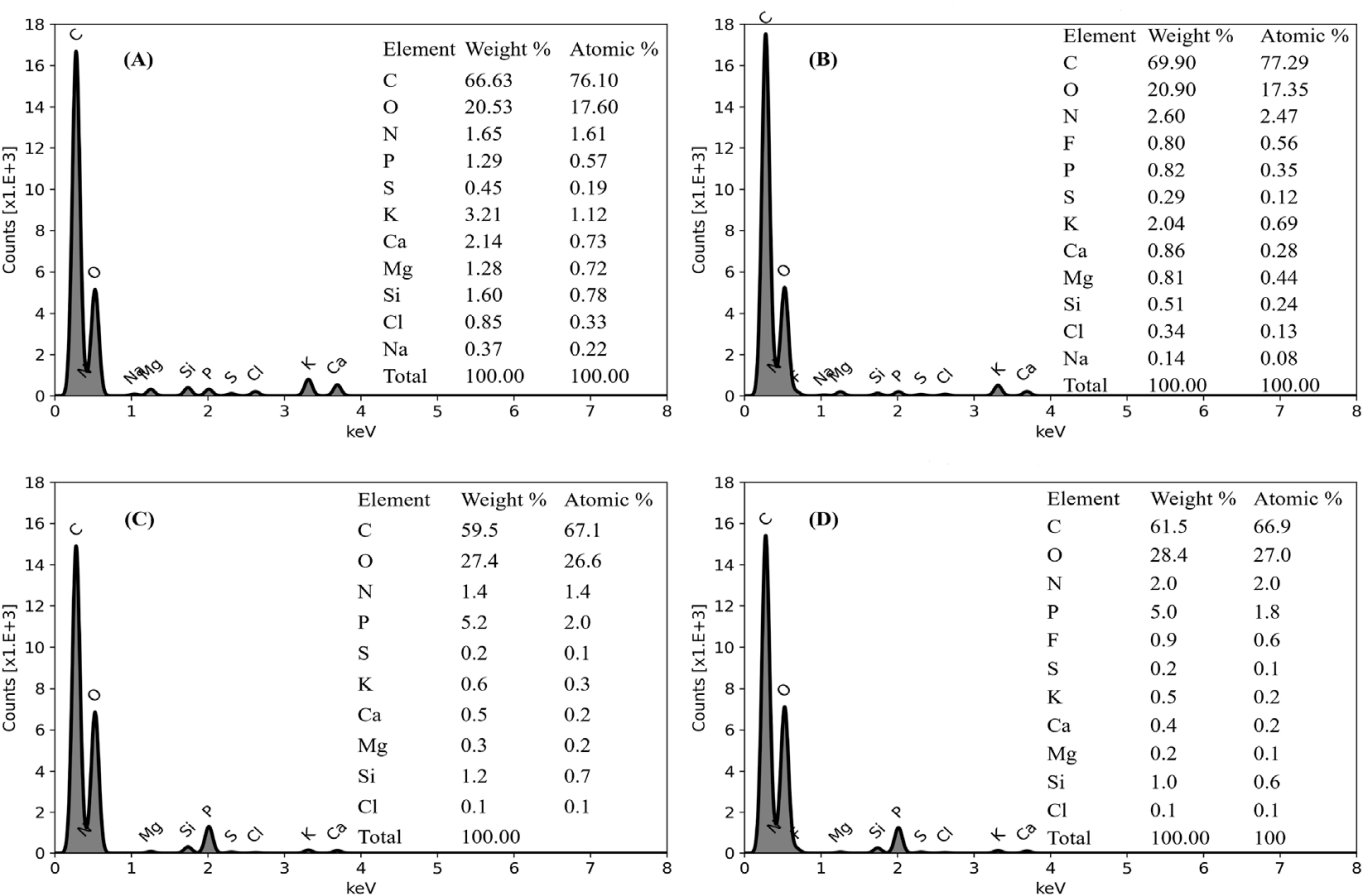
EDX analysis of CLB10 before adsorption **(A)**, after adsorption **(B)**, PAM-CLB1.5 before adsorption **(C)**, and after adsorption **(D)**.

The EDX spectra, as shown in Figures 3A,B, revealed that after CIP adsorption, the nitrogen content in CLB10 was increased from 1.65 wt% to 2.60 wt%, and fluorine, initially absent, appears at 0.80 wt% in (B), directly confirming CIP adsorption on the biochar. Carbon and oxygen also rose from 66.63 wt% to 69.90 wt% and 20.53 wt% to 20.90 wt%, respectively. The increased C and O contents after adsorption are attributed to the successful loading of ciprofloxacin (CIP) onto the biochar surface. The CIP contains a carbon-rich aromatic structure and oxygen-containing functional groups (carboxyl, carbonyl, hydroxyl), which contribute additional C and O signals in EDX analysis after adsorption (Iqbal et al., 2022).

While potassium and calcium decrease (K drops from 3.21 wt% to 2.04 wt%, Ca from 2.14 wt% to 0.86 wt%), supporting that cation exchange occurs during adsorption.

Phosphoric acid modification (PAM-CLB1.5) oxidized the biochar as shown in Figure 3C, decreasing carbon content from 66.63 to 59.5 wt% and increasing oxygen from 20.53 wt% to 27.4 wt% due to new oxygenated groups. Phosphorus was increased from 1.29 wt% to 5.2 wt%, confirming phosphate functionalization (C–O–P, P–O). Acid leaching lowered alkali metals (K, Ca, Mg, Na), reducing ash, while nitrogen content remained nearly unchanged, indicating stable N-groups. PAM-CLB1.5 in Figure 3C,D showed elevated oxygen (27.4 wt%) and phosphorus (5.2 wt%), with concentrations dropping after CIP uptake (C rises from 59.5 wt% to 61.5 wt%, O from 27.4 wt% to 28.4 wt%), and similar increases in N and F content. These changes validate the roles of functional groups and cation exchange in CIP removal. The increased C, O, N, and F signals after adsorption further support CIP loading onto the biochar surface, while phosphorus enrichment after modification confirms phosphate functionalization, linking elemental changes with improved adsorption affinity.

#### 3.1.4 BET

The BET surface area of CLB10 was found as 15.0327 m^2^/g (Table 4), which is higher than reported mango peel biochar (Zhang et al., 2020); rice husk biochar (Liu et al., 2025); spent mushroom substrate biochar (Xian et al., 2018); halophyte-based biochar (Wang et al., 2022); and melinjo seed shell biochar (Rahmat et al., 2024); i.e., 5.74 m^2^ g^-1^, 5.40 m^2^ g^-1^, 12.97 m^2^ g^-1^, 11.89 m^2^ g^-1^ and 4.10 m^2^ g^-1^, respectively. The pore volume of CLB10 in the current study was found to be 0.06418cm^3^ g^-1^, which is also higher than that of mango peel biochar (Zhang et al., 2020); rice husk biochar (Liu et al., 2025); and halophyte-based biochar (Wang et al., 2022); i.e., 0.00182 cm^3^ g^-1^, 0.0098 cm^3^ g^-1^, and 0.0291cm^3^ g^-1^, respectively. The pore size of CLB10, as mentioned in Table 4, was observed as 4.593 nm, which is greater than other reported biochars in literature, such as mango peel biochar (Zhang et al., 2020); and rice husk biochar (Liu et al., 2025); i.e., 3.49 nm and 4.43 nm, respectively. The BET surface area, pore volume and pore size of phosphoric acid modified CLB10, i.e., PAM-CLB1.5 is reported (Table 4) higher than CLB10 in current study as 24.6541m^2^/g, 0.1542 cm^3^/g and 5.755 nm, respectively which is also higher than many modified biochars in literature like H_3_PO_4_-modified corn-stalk biochar shows a BET surface area of 3 m^2^ g^-1^, pore volume 0.0025 cm^3^ g^-1^, and pore diameter 3.51 nm (Liu et al., 2019). Similarly, NaOH- and EDTA-modified poplar-branch biochars exhibit a surface area of 3.66 and 1.64 m^2^ g^-1^, pore volumes of 0.002 and 0.005 cm^3^ g^-1^, and pore sizes of 9.15 nm and 12.27 nm, respectively (Liu et al., 2022). Additionally, HCl-modified rice-straw biochar displays a BET surface area of 1.508 m^2^ g^-1^, pore volume 0.003 cm^3^ g^-1^, and pore diameter 1.53 nm (Cera et al., 2024). Collectively, these findings confirm that CLB10 and PAM-CLB1.5 possess a more enhanced porous structure compared with most raw and modified biochars in the literature.

The data in Table 4 show that PAM-CLB1.5 exhibits higher BET surface area, increased pore volume, and a larger average pore size. This enhancement results from the chemical activation by phosphoric acid that introduces phosphate functional groups on the biochar surface, which contributes to increased surface polarity and provides active sites for chemical interactions (Jiang et al., 2024).

These structural changes directly enhance adsorption efficiency by increasing the accessible surface area for adsorbate molecules and improving mass transfer through expanded pore networks (Ries et al., 2025). Studies have shown that phosphoric acid modification leads to simultaneous physical adsorption within micropores and chemisorption on functional groups, making the biochar more effective in pollutant removal. The enhancement is more reasonably attributed to the combined increase in pore volume from 0.064 to 0.154 cm^3^ g^-1^, increased pore size from 4.593 to 5.755 nm, and improved surface accessibility after H_3_PO_4_ modification. Similar findings have been reported for acid-modified biochar, where superior antibiotic removal was governed by synergistic effects of porosity and acidic oxygen-containing functional groups rather than the highest surface area alone.

#### 3.1.5 Determination of pH_p_zc

**FIGURE 4:**
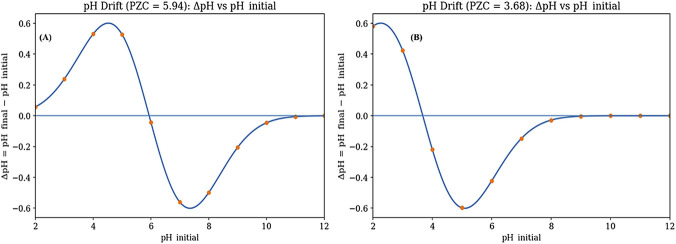
pH_p_zc analysis of CLB10 **(A)** and PAM-CLB1.5 **(B)**.

The point of zero charge (pH_p_zc) is fundamental to understanding CIP adsorption on biochar as a function of pH. Results in Figure 4A show that in the case of CLB10 the pH_p_zc value is 5.94, so the CIP removal increases near neutral pH, where the biochar surface acquires a negative charge (pH > pH_p_zc) and attracts the zwitterionic or cationic forms of CIP via electrostatic interactions and hydrogen bonding.

In contrast, PAM-CLB1.5 biochar, modified with phosphoric acid (pH_p_zc 3.68), shows a greater negative surface charge at lower pH, enabling efficient CIP removal across a broader pH range. Phosphoric acid (H_3_PO_4_) modification consistently lowers the pH_p_zc of biochars as shown in Figure 4B, thereby enhancing their surface acidity and increasing their negative charge density. For example, the pH_p_zc of cauliflower leaves biochar used for Cr (II) and Pb (II) removal declined significantly from 5.84 to 3.27 following H_3_PO_4_ impregnation (Ge et al., 2020c). Likewise, H_3_PO_4_-activated eucalyptus biochar demonstrated a pH_p_zc of approximately 3.9 (Ries et al., 2025). In a similar trend, phosphorus-rich biochar derived from chicken feathers exhibited a reduced pH_p_zc of 3.53 compared with 5.53 for the unmodified biochar (Chen et al., 2020). The pH_p_zc decreased from 5.94 for CLB10 to 3.68 for PAM-CLB1.5, indicating a clear shift toward increased surface acidity following H_3_PO_4_ treatment. This decrease can be attributed to the introduction of oxygenated and phosphorus-containing acidic functionalities, including phosphate-related surface groups generated during chemical activation. As a consequence, the modified biochar exhibits a negative surface charge over a broader pH range compared with the raw biochar, potentially enhancing electrostatic interactions with CIP species. Optimal pH for CIP adsorption coincides as discussed in Section 3.4.1.2 with maximum negative surface charge and CIP’s zwitterionic form, thus biochar’s pH_p_zc and pH directly regulate removal efficiency through surface charge and molecular speciation effects (Ouyang et al., 2023). These findings are consistent with FTIR and EDX results demonstrating altered surface chemistry after phosphoric acid modification.

### 3.2 Box-Behnken Design (BBD) and model analysis for biochar preparation

According to the RSM statistical matrix, 17 runs were generated to set up the experimental design. The variables used in the experimental design and their predicted and actual results are displayed in Table 5. The CIP removal efficiencies, both actual and predicted by the quadratic model, show close agreement, indicating a well-fitted model. Removal percentages ranged from a minimum of 47.78% (at the shortest time, lowest temperature, and medium particle size) to a maximum of 67.06% (at moderate time and temperature, with medium particle size). The data reveal that increasing pyrolysis time and temperature generally enhances CIP adsorption, while smaller particle sizes tend to improve removal efficiency due to increased surface area. Overall, the BBD effectively plotted the combined impacts of these factors on CIP removal, supporting optimization of biochar preparation conditions for enhanced adsorption performance.

**TABLE 5:** Box behnken design for biochar preparation.

Run	Factor 1 A. Time (min)	Factor 2 B. Temperature (°C)	Factor 3 C. Particle size (μm)	CLB10	
				CIP removal %	
				Actual	Predicted
1	60	475	125	57.58	57.87
2	90	475	272.5	65.82	66.43
3	120	300	272.5	53.34	53.49
4	120	475	420	61.42	61.13
5	90	475	272.5	66.23	66.43
6	90	300	420	57.34	57.48
7	60	475	420	55.26	55.19
8	90	475	272.5	66.98	66.43
9	120	650	272.5	57.18	57.24
10	90	475	272.5	67.06	66.43
11	60	650	272.5	52.87	52.72
12	90	300	125	59.38	59.16
13	90	475	272.5	66.05	66.43
14	90	650	420	61.42	61.65
15	60	300	272.5	47.78	47.72
16	120	475	125	62.15	62.22
17	90	650	125	63.88	63.74

The regression coefficients (Table 6) indicate how each factor, pyrolysis time, temperature, and particle size, affects CIP removal efficiency using CLB10. The positive coefficients for time (2.58) and temperature (2.19), as mentioned in Table 6, showed that increasing these parameters enhances CIP removal, while the negative coefficient for particle size (−0.94) means that smaller particles slightly improve removal efficiency. The significant quadratic terms for time (−7.52) and temperature (−6.12) highlight that their effect on CIP removal is nonlinear, with optimal conditions likely at intermediate levels. Interaction terms, such as AC and AB, are less significant, implying limited combined effects between factors. Overall, the model demonstrates a good fit, with high significance and predictive power, indicating that optimizing pyrolysis time and temperature while managing particle size can significantly improve adsorptive removal of CIP.

**TABLE 6:** Regression coefficients of the independent terms for Biochar Preparation.

Factor	Coefficient estimate	df	Standard error	95%CI low	95%CI high	VIF
Intercept	66.43					
A-Time	2.58	1	0.1704	2.17	2.98	1.0000
B-Temperature	2.19	1	0.1704	1.79	2.59	1.0000
C-Particle Size	−0.9437	1	0.1704	−1.35	−0.5408	1.0000
AB	−0.3125	1	0.2410	−0.8824	0.2574	1.0000
AC	0.3975	1	0.2410	−0.1724	0.9674	1.0000
BC	−0.1050	1	0.2410	−0.6749	0.4649	1.0000
A^2^	−7.52	1	0.2349	−8.07	−6.96	1.01
B^2^	−6.12	1	0.2349	−6.67	−5.56	1.01
C^2^	0.1935	1	0.2349	−0.3620	0.7490	1.01

Table 7 evaluates the BBD quadratic model for CIP removal by CLB10. The model is highly significant (F = 247.72, p < 0.0001). Among factors, pyrolysis time (A) and temperature (B) significantly influence removal with large F-values (228.30 and 164.94) and p-values <0.0001 as given in Table 7. Particle size (C) also affects removal significantly but to a lesser extent (F = 30.67, p = 0.0009). Interaction terms (AB, AC, BC) were not significant (p > 0.1). Quadratic terms for time (A^2^) and temperature (B^2^) are significant (p < 0.0001), indicating nonlinear effects. The model residual error is low (Std. Dev. = 0.482), and lack of fit is not significant (p = 0.7653), confirming good model fit. Adequate precision at 50.61 indicates a strong signal-to-noise ratio, supporting model reliability for optimizing CIP removal. The coefficient of variation (0.802%) confirms high experimental precision. Impact of pyrolysis time, temperature, and particle size is A > B > C as shown in Equation 8.

$$\text{CIP Removal}\%=-58.4354+1.59343\;*A+0.208709\;*\;B-0.0173981\;*\;C-5.95238e-05\;*\text{ AB}+8.98305e-05\;*\text{AC}-4.0678e-06\;*\text{ BC}-0.00835444\;*\;A^{2}-0.000199722\;*\;B^{2}+8.894e-06\;*\;C^{2}$$ (8)

**TABLE 7:** ANOVA for Quadratic model for Biochar preparation.

Source	Sum of squares	df	Mean square	F-value	p-value	​
Model	518.01	9	57.56	247.72	<0.0001	significant
A-Time	53.05	1	53.05	228.30	<0.0001	​
B-Temperature	38.33	1	38.33	164.94	<0.0001	​
C-Particle Size	7.13	1	7.13	30.67	0.0009	​
AB	0.3906	1	0.3906	1.68	0.2359	​
AC	0.6320	1	0.6320	2.72	0.1431	​
BC	0.0441	1	0.0441	0.1898	0.6762	​
A^2^	238.04	1	238.04	1024.50	<0.0001	​
B^2^	157.52	1	157.52	677.95	<0.0001	​
C^2^	0.1577	1	0.1577	0.6785	0.4373	​
Residual	1.63	7	0.2324	​	​	​
Lack of Fit	0.3706	3	0.1235	0.3934	0.7653	not significant
Pure Error	1.26	4	0.3140	​	​	​
Cor Total	519.64	16	​	​	​	​

#### 3.2.1 Effect of biochar preparation conditions on CIP adsorption

##### 3.2.1.1 Pyrolysis time

**FIGURE 5:**
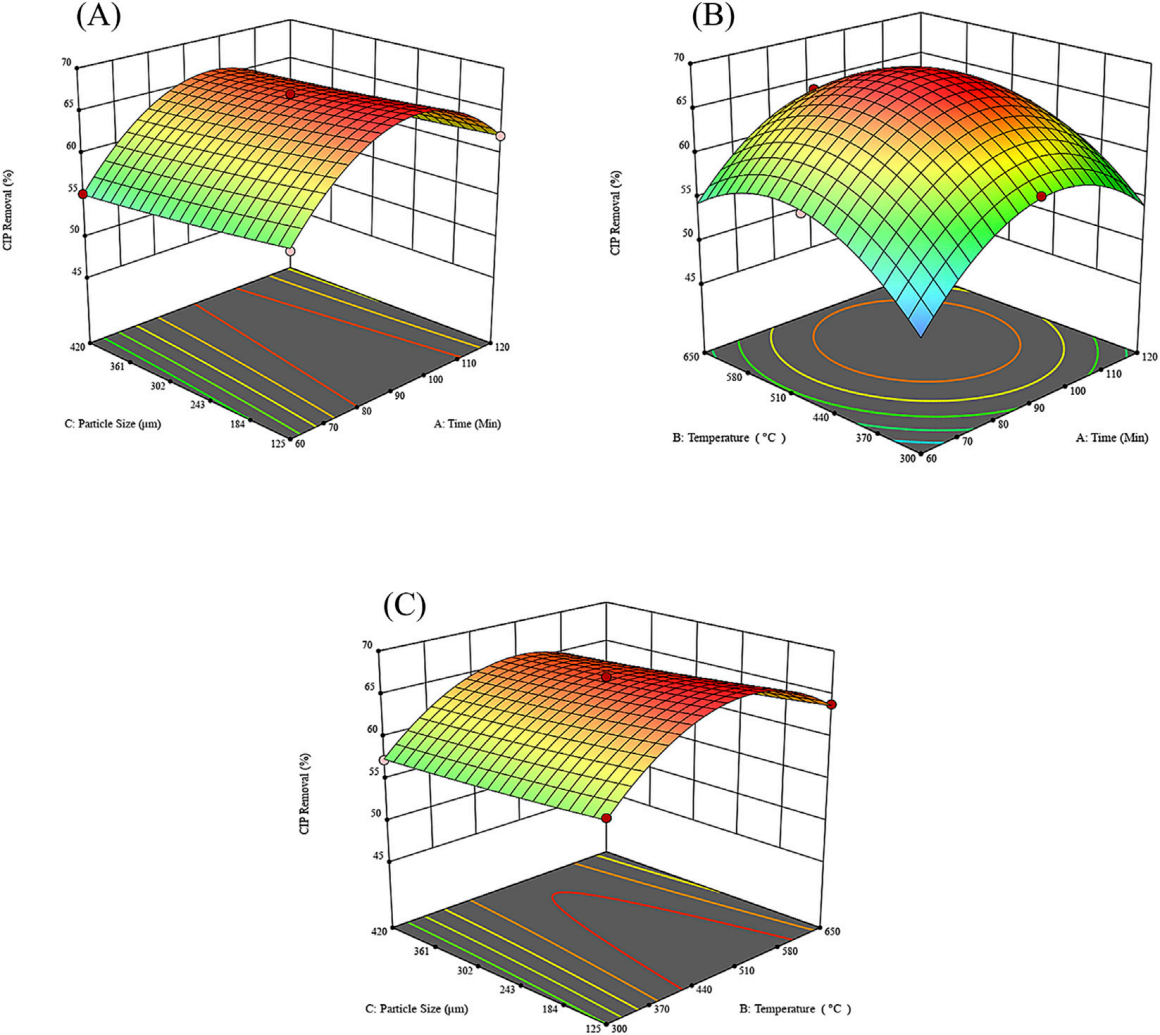
Impact of particle size and Time **(A)** Temperature and Time **(B)**, and Particle size and temperature **(C)** on the CIP removal%.

Pyrolysis time influences properties of biochar such as surface area, porosity, functional groups, and adsorption capacity. Results in the Figures 5A,B show that CIP removal efficiency is altered with a change in pyrolysis time, while maintaining constant temperature (B = 475 °C) and particle size (C = 125 µm). The trend is a parabolic (quadratic) trend, meaning that the removal of CIP initially increases with time but decreases beyond a certain optimal value (90 min). CLB10 showed a decline in adsorption efficiency after reaching the optimal pyrolysis time. CLB10 exhibited a maximum removal efficiency of 66.98% at 90 min, which declined to 62.22% at 120 min. Similar trends have been reported for the effect of pyrolysis time on biochar adsorption performance by many researchers (Al-Bayati et al., 2024; Sun et al., 2017; Sun et al., 2025). Ouyang et al. (2023) observed a decrease in methylene blue removal from 47.5% to 30.3% when pyrolysis time increased from 30 to 90 min at constant temperature. Sun et al. (2025) reported that Pb (II) removal increased with pyrolysis time, obtained a maximum value at 120 min (96.7% removal), and declined thereafter. Zuo et al. (2024) identified an optimal pyrolysis time of 120 min for corncob biochar, achieving 73.55% Cr (VI) removal. This adsorption trend is also reported in a study about the use of biochar to remove pharmaceutical compounds (Ahmad et al., 2014). Increased time of pyrolysis increases the pore structure of biochar, raising its surface area, as well as microporosity (Peng et al., 2011). However, extending the pyrolysis duration beyond the optimal point leads to the collapse of pore structures, depletion of oxygen-containing functional groups that are essential for adsorption, and accumulation of inorganic ash and mineral residues that cause pore blockage. Collectively, these effects contribute to a significant reduction in the overall adsorption efficiency of the biochar (Ouyang et al., 2023).

##### 3.2.1.2 Pyrolysis temperature

Pyrolysis temperature significantly impacts the adsorption efficiency of biochar by altering its physicochemical properties. Increasing pyrolysis temperature generally enhances the biochar’s surface area, porosity, and aromaticity, which contribute to improved adsorption capacity (Li et al., 2023). Removal of CIP improved as shown in the Figures 5B,C with the increase in temperature up to an optimum value (approximately 475 °C), then decreased with further increase in temperature, suggesting that higher pyrolysis temperatures enhance biochar porosity and surface functionality, leading to improved adsorption capacity and CIP removal efficiency as illustrated in Figures 5B,C. However, beyond the optimal temperature, excessive thermal degradation reduces the biochar’s adsorption sites, causing a decline in performance. CLB10 showed a maximum removal of 66.98% at 475 °C, which dropped to 63.74% at 650 °C, indicating a reduction in adsorption performance with high pyrolysis temperature due to deoxygenation of polar oxygen-containing functional groups, collapse of micropores, decreasing in BET surface area, and pore blockage due to higher ash production (Murtaza et al., 2022). Several studies show that biochar adsorption performance does not increase monotonically with pyrolysis temperature. Akhtar et al. (Akhtar et al., 2021) reported substantially higher fluoroquinolone removal at a low temperature of 300 °C than at 700 °C, indicating performance loss at high temperatures. Similarly, Mabaso et al. (2024) achieved near-complete Pb (II) removal using a mid-temperature biochar (475 °C).

##### 3.2.1.3 Particle size

The biomass particle size before pyrolysis has a great influence on the adsorption efficiency of resultant biochar. Small particles have enhanced specific surface areas and greater microporosity, resulting in more uniform heat transfer and release of volatiles during pyrolysis (Jia et al., 2018; Yaashikaa et al., 2020). A decrease in CIP removal efficiency with increasing biomass particle size is observed in the current study, as shown in Figures 5A,C. A slight decrease in CIP removal efficiency was observed, from 66.98% to 65.67%, as particle size was increased, suggesting that biochar derived from smaller biomass particles exhibits higher adsorption performance. A reduction in biomass particle size before pyrolysis significantly improved adsorption uptake. This enhancement was attributed to more uniform heat transfer and improved pore development during the pyrolysis of smaller biomass particles (Jia et al., 2018). Similar results were observed by Madariaga et al. (Madariaga-Segovia et al., 2025) for the removal of paracetamol from water using hazelnut shell biochar. This is because smaller particles, upon pyrolysis, tend to develop a larger surface area and more active sites, enhancing their ability to adsorb CIP compared to biochar produced from larger biomass particles. Another reason is that smaller biomass particles ensure more uniform heat distribution during pyrolysis, leading to biochar with enhanced textural properties such as high surface area and microporosity (He et al., 2022). The chemical structure of biochar is also affected by biomass size, where smaller particles may lead to higher aromaticity and better surface functionalities that contribute to adsorption efficiency.

### 3.3 Chemical modification

**FIGURE 6:**
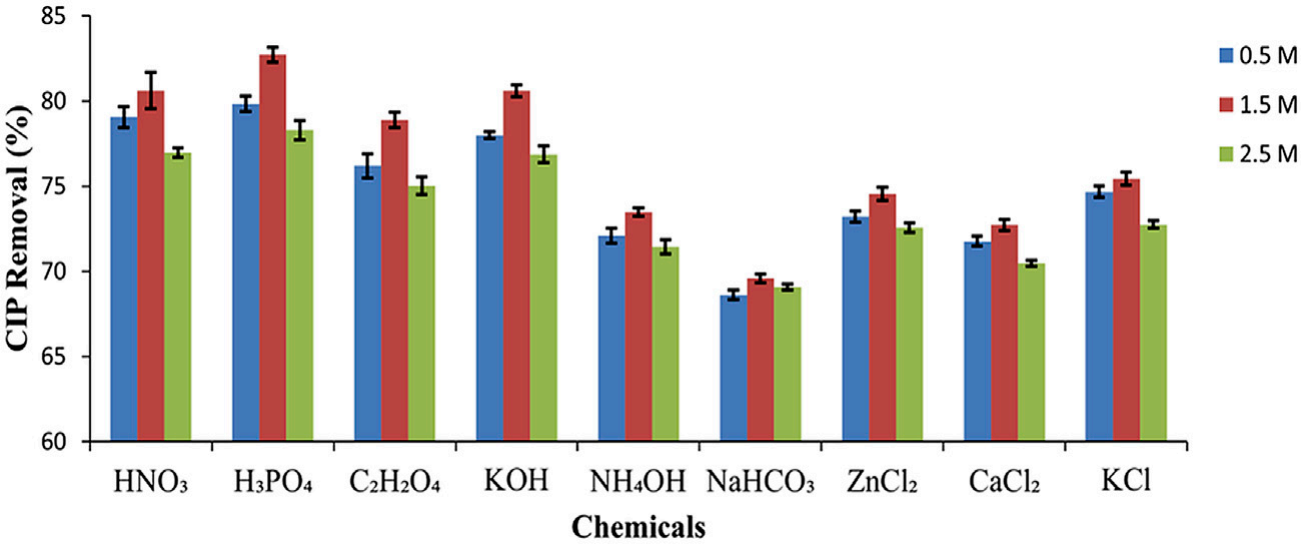
Effect of chemical modification and reagent concentration on CIP removal efficiency (%).

Selected biochar, i.e., CLB15 (Supplementary Table A1), with the lowest removal efficacy of 47.78% (at the shortest time, lowest temperature, and medium particle size), was modified with different chemicals and concentrations as discussed in Section 2.4 and enlisted in Table 2 that could be attributed to the modifications in the physicochemical properties of biochar induced by the chemical treatments. The concentration range selected for chemical modification (0.5–2.5 M) was based on literature guidance, together with preliminary screening experiments conducted prior to the detailed adsorption study. Preliminary observations suggested that concentrations below 0.5 M resulted in comparatively limited enhancement of biochar adsorption behavior, indicating insufficient surface modification (Iqbal et al., 2022). In contrast, concentrations exceeding 2.5 M did not provide proportionally significant improvement in adsorption performance and may contribute to excessive chemical usage, over-activation, or undesirable alteration of biochar structure (Liu et al., 2019; Liu et al., 2022). Therefore, the selected concentrations (0.5, 1.5, and 2.5 M) were considered suitable for systematically investigating the influence of modification intensity on biochar physicochemical properties and ciprofloxacin adsorption performance while maintaining adsorbent structural stability. Results in Figure 6 show that phosphoric acid modification exhibits the highest removal efficiency, particularly at 1.5 M concentration (PAM-CLB1.5), which can be due to the introduction of acidic functional groups and increased porosity that enhance adsorption sites and interaction mechanisms such as hydrogen bonding and electrostatic attraction with CIP molecules (Farhaneem et al., 2018). Similarly, nitric acid and potassium hydroxide modifications (Figure 6) improve surface area and porosity, creating more active sites, but their effects plateau or slightly decrease at higher concentrations due to possible pore blockage or saturation of surface sites (Inyang et al., 2015). Oxalic acid and zinc chloride bring moderate improvements by modifying surface chemistry, while chemicals like ammonium hydroxide, sodium bicarbonate, and calcium chloride result in lower removal efficiencies, likely due to weaker surface modification and fewer functional groups available for adsorption (Murtaza et al., 2024). The maximum CIP removal observed at 1.5 M in all cases of chemical modification indicates that intermediate modification strength provides an optimum balance between effective surface activation and preservation of biochar structure. Lower concentration (0.5 M) may be insufficient to significantly improve pore structure, surface area, mineral composition, or active functional groups, resulting in limited adsorption enhancement. In contrast, higher concentration (2.5 M) can cause over-activation, excessive oxidation, pore blockage/widening, mineral leaching, and partial loss or masking of reactive sites, thereby reducing adsorption efficiency. Thus, 1.5 M provides the most favorable conditions for enhancing physicochemical properties and the adsorbent–adsorbate interactions governing CIP uptake, consistent with previous studies reporting superior performance under moderate chemical activation conditions (Liu et al., 2020). This interplay of surface chemistry, porosity, and functional groups governs the adsorption capacity of modified biochar for CIP removal in aqueous solutions.

### 3.4 Box-Behnken Design (BBD) and model analysis for batch experiments

Based on the RSM statistical matrix, a total of 30 experimental runs were generated to establish the experimental design. The variables included in the design, along with their predicted and actual responses, are presented in Table 8. The experiments varied four factors: contact time (A), pH (B), CIP concentration (C), and adsorbent dose (D). Results in Table 8 showed that PAM-CLB1.5 performed far better than CLB10 in CIP removal, reflecting the impact of phosphoric acid modification, which enhances surface area, pore volume, and introduces more functional groups that improve adsorption (Alawa et al., 2025).

**TABLE 8:** Box behnken design for CIP removal % for CLB10 and PAM-CLB1.5.

Run	Factor 1 A. Time (min)	Factor 2	Factor 3 C. CIP Concentration (mg/L)	Factor 4 D. Adsorbent dose (mg)	CLB10		PAM-CLB1.5	
					Removal%		Removal%	
					Actual	Actual	Predicted	Predicted
1	70	12	50	30	50.58	50.93	61.47	60.66
2	120	2	30	30	56.43	56.79	63.73	64.11
3	70	2	50	30	54.41	54.28	59.86	59.68
4	20	7	30	50	60.93	60.80	73.89	73.88
5	70	7	10	10	64.35	64.85	82.68	82.64
6	70	7	30	30	65.9	65.77	75.37	77.22
7	70	2	30	50	61.52	61.85	71.36	71.25
8	70	7	30	30	66.42	65.77	77.82	77.22
9	120	12	30	30	53.74	53.89	63.74	64.41
10	70	7	30	30	64.73	65.77	78.46	77.22
11	70	7	30	30	66.82	65.77	77.21	77.22
12	20	7	30	10	48.34	48.52	56.58	57.06
13	70	12	30	10	46.75	46.26	56.12	56.55
14	120	7	30	10	57.48	57.39	71.35	70.79
15	20	12	30	30	44.59	44.62	52.63	52.50
16	70	7	10	50	77.35	77.64	96.14	96.79
17	70	12	30	50	58.72	58.76	72.97	73.19
18	120	7	10	30	57.48	57.39	90.07	89.95
19	120	7	30	50	70.69	70.28	86.66	85.62
20	70	2	10	30	64.65	64.07	74.87	75.12
21	70	12	10	30	61.52	61.43	76.78	76.40
22	70	7	30	30	65.26	65.77	75.54	77.22
23	70	7	30	30	65.49	65.77	78.93	77.22
24	70	7	50	50	67.41	67.29	82.59	82.88
25	70	2	30	10	49.38	49.18	56.14	56.23
26	20	2	30	30	47.49	47.72	50.96	50.54
27	70	7	50	10	54.81	54.91	65.77	65.37
28	120	7	50	30	62.35	62.32	73.11	73.78
29	20	7	50	30	53.53	53.36	61.19	61.63
30	20	7	10	30	48.34	48.52	76.98	76.63

Across runs under different factor combinations, PAM-CLB1.5’s removal percentages range from about 50.93% to 96.79%, substantially higher than the corresponding CLB10 range of 44.59%–77.64%, indicating the modification greatly boosts adsorption capacity and efficiency. The predicted removal values from the RSM model closely match the actual values, demonstrating the model’s robustness and accuracy in optimizing the adsorption process.

The coefficient estimates in Table 9 for CIP removal by CLB10 and PAM-CLB1.5 from the RSM highlights important factor effects on adsorption performance. For CLB10, time (A) positively influences removal (coefficient 4.59), indicating longer contact enhances adsorption. pH (B) has a significant negative effect (−1.50), showing removal decreases as pH deviates from the optimal range. CIP concentration (C) negatively impacts removal (−5.07), reflecting site saturation at higher pollutant loads. Adsorbent dose (D) positively affects removal (6.29), consistent with more active sites improving efficiency. Quadratic terms of time (A^2^) and pH (B^2^) are significantly negative (−4.89 and −10.13), revealing optimum conditions beyond which effects decline, while the positive quadratic term for concentration (C^2^ = 2.03) indicates increasing levels have a stabilizing effect.

**TABLE 9:** Regression coefficients of the independent terms for CIP removal.

Factor	Coefficient estimate	df	Standard error	95%CI low	95%CI high	VIF
CLB10						
Intercept	65.77	1	0.2277	65.28	66.26	​
A-Time	4.59	1	0.1610	4.24	4.93	1.0000
B-pH	−1.50	1	0.1610	−1.84	−1.16	1.0000
C-CIP Concentration	−5.07	1	0.1610	−5.42	−4.73	1.0000
D-Adsorbent Dose	6.29	1	0.1610	5.95	6.64	1.0000
AB	0.0525	1	0.2789	−0.5419	0.6469	1.0000
AC	−0.1025	1	0.2789	−0.6969	0.4919	1.0000
AD	0.1550	1	0.2789	−0.4394	0.7494	1.0000
BC	−0.1750	1	0.2789	−0.7694	0.4194	1.0000
BD	−0.0425	1	0.2789	−0.6369	0.5519	1.0000
CD	−0.1000	1	0.2789	−0.6944	0.4944	1.0000
A^2^	−4.89	1	0.2130	−5.34	−4.44	1.05
B^2^	−10.13	1	0.2130	−10.58	−9.67	1.05
C^2^	2.03	1	0.2130	1.58	2.49	1.05
D^2^	−1.63	1	0.2130	−2.09	−1.18	1.05
PAM-CLB1.5						
Intercept	77.22	1	0.4223	76.32	78.12	​
A-Time	6.37	1	0.2986	5.73	7.01	1.0000
B-pH	0.5658	1	0.2986	−0.0706	1.20	1.0000
C-CIP Concentration	−7.79	1	0.2986	−8.43	−7.16	1.0000
D-Adsorbent Dose	7.91	1	0.2986	7.28	8.55	1.0000
AB	−0.4150	1	0.5172	−1.52	0.6873	1.0000
AC	−0.2925	1	0.5172	−1.39	0.8098	1.0000
AD	−0.5000	1	0.5172	−1.60	0.6023	1.0000
BC	−0.0750	1	0.5172	−1.18	1.03	1.0000
BD	0.4075	1	0.5172	−0.6948	1.51	1.0000
CD	0.8400	1	0.5172	−0.2623	1.94	1.0000
A^2^	−5.90	1	0.3950	−6.74	−5.06	1.05
B^2^	−13.43	1	0.3950	−14.28	−12.59	1.05
C^2^	4.18	1	0.3950	3.33	5.02	1.05
D^2^	0.5187	1	0.3950	−0.3231	1.36	1.05

In case of PAM-CLB1.5, chemical modification enhanced the influence of the operating variables on adsorption performance. Among these variables, contact time (6.37) and adsorbent dose (7.91) showed the greatest positive effects on CIP removal. The pH coefficient becomes positive but less certain, indicating possible shifts in optimum pH due to surface chemistry changes. The negative concentration effect (−7.79) remains strong. Quadratic terms A^2^ (−5.90) and B^2^ (−13.43) again show pronounced curvature effects. The lower magnitude and some positive interaction terms suggest PAM-CLB1.5’s improved and more complex surface interactions with CIP.

The ANOVA results as shown in the Table 10 for both CLB10 and PAM-CLB1.5 models indicate statistically significant overall models (p < 0.0001). For CLB10, the model sum of squares is 1958.66 with 14 degrees of freedom (df), having mean square and F-value of 139.90 and 449.79, respectively. Significant factors include A-Time, B-pH, C-CIP concentration, and D-Adsorbent Dose (all p < 0.0001). Only quadratic terms are significant among interactions (p < 0.0001). Residual sum of squares is 4.67 (15 df), and lack of fit is non-significant (p = 0.9557), showing good model fit.

**TABLE 10:** ANOVA for Quadratic model for CIP removal%.

Source	Sum of squares	df	Mean square	F-value	p-value	​
CLB10						
Model	1958.66	14	139.90	449.79	<0.0001	significant
A-Time	252.45	1	252.45	811.62	<0.0001	​
B-PH	26.94	1	26.94	86.61	<0.0001	​
C-CIP Concentration	308.76	1	308.76	992.66	<0.0001	​
D-Adsorbent Dose	475.15	1	475.15	1527.58	<0.0001	​
AB	0.0110	1	0.0110	0.0354	0.8532	​
AC	0.0420	1	0.0420	0.1351	0.7183	​
AD	0.0961	1	0.0961	0.3090	0.5865	​
BC	0.1225	1	0.1225	0.3938	0.5397	​
BD	0.0072	1	0.0072	0.0232	0.8809	​
CD	0.0400	1	0.0400	0.1286	0.7249	​
A^2^	164.00	1	164.00	527.24	<0.0001	​
B^2^	703.02	1	703.02	2260.19	<0.0001	​
C^2^	28.35	1	28.35	91.15	<0.0001	​
D^2^	18.26	1	18.26	58.69	<0.0001	​
Residual	4.67	15	0.3110	​	​	​
Lack of Fit	1.70	10	0.1704	0.2876	0.9557	not significant
Pure Error	2.96	5	0.5924	​	​	​
Cor Total	1963.32	29	​	​	​	​
PAM-CLB1.5						
Model	3669.32	14	262.09	244.99	<0.0001	significant
A-Time	486.80	1	486.80	455.03	<0.0001	​
B-PH	3.84	1	3.84	3.59	0.0775	​
C-CIP Concentration	728.99	1	728.99	681.41	<0.0001	​
D-Adsorbent Dose	751.61	1	751.61	702.56	<0.0001	​
AB	0.6889	1	0.6889	0.6439	0.4348	​
AC	0.3422	1	0.3422	0.3199	0.5800	​
AD	1.0000	1	1.0000	0.9347	0.3490	​
BC	0.0225	1	0.0225	0.0210	0.8866	​
BD	0.6642	1	0.6642	0.6209	0.4430	​
CD	2.82	1	2.82	2.64	0.1251	​
A^2^	238.80	1	238.80	223.21	<0.0001	​
B^2^	1237.48	1	1237.48	1156.72	<0.0001	​
C^2^	119.60	1	119.60	111.79	<0.0001	​
D^2^	1.85	1	1.85	1.72	0.2088	​
Residual	16.05	15	1.07	​	​	​
Lack of Fit	4.98	10	0.4981	0.2250	0.9784	not significant
Pure Error	11.07	5	2.21	​	​	​
Cor Total	3685.36	29	​	​	​	​

While in case of PAM-CLB1.5, the model sum of squares is 3669.32 with 14 df, mean square 262.09, and F-value 244.99 (p < 0.0001). Significant factors are A-Time, C-CIP Concentration, D-Adsorbent Dose, and quadratic terms A^2^, B^2^, and C^2^ (p < 0.0001). B-pH and D^2^ and all interactions were not significant. Residual sum of squares is 16.05 (15 df) with non-significant lack of fit (p = 0.9784), indicating a good fit. Total sum of squares is 1963.32 for CLB10 and 3685.36 for PAM-CLB1.5 with 29 total df.

These findings show strong effects of time, concentration, and dose on adsorption, with several quadratic effects significant, reflecting the nonlinear relationships in biochar adsorption behavior. The non-significant lack of fit confirms appropriate model adequacy for both biochar.

$$\text{CIP Removal }\%\text{ by CLB}10=30.6204+0.362552\;*\;A+5.42112\;*\;B-0.5317\;*\;C+0.559\;*\;D+0.00021\;*\text{ AB}-0.0001025\;*\text{ AC}+0.000155\;*\text{ AD}-0.00175\;*\text{ BC}-0.000425\;*\text{ BD}-0.00025\;*\text{CD}-0.00195617\;*\;A^{2}-0.405017\;*\;B^{2}+0.00508333\;*\;C^{2}-0.00407917\;*\;D^{2}$$ (9)

$$\text{CIP Removal }\%\text{ by PAM}-\text{CLB}1.5=40.1104+0.493248\;*\;A+7.65252\;*\;B-1.05342\;*\;C+0.261371\;*\;D-0.00166\;*\text{ AB}-0.0002925\;*\text{ AC}-0.0005\;*\text{ AD}-0.00075\;*\text{ BC}+0.004075\;*\text{ BD}+0.0021\;*\text{ CD}-0.0023605\;*\;A^{2}-0.53735\;*\;B^{2}+0.0104406\;*\;C^{2}+0.00129687\;*\;D^{2}$$ (10)

The Equations 9, 10 for CIP removal (%) by CLB10 and its phosphoric acid modified counterpart PAM-CLB1.5, describe the interactive effects of process variables using a quadratic polynomial model.

For CLB10, the equation shows that time (A) and pH (B) positively contribute to removal (coefficients 0.3626 and 5.4211, respectively), while initial CIP concentration (C) has a negative impact (−0.5317), indicating a decrease in removal efficiency with increased pollutant load. Adsorbent dose (D) also positively influences removal (0.559). The interaction and quadratic terms have relatively smaller coefficients, indicating moderate curvature and interaction effects. On the other hand, PAM-CLB1.5 exhibits a generally stronger influence of time (0.4932) and pH (7.6525) on removal efficiency than CLB10, while CIP concentration remains a strong negative factor (−1.0534). Adsorbent dose has a positive but lower coefficient (0.261) compared to CLB10. Quadratic and interaction terms suggest more complex relationships in the modified biochar system, likely due to enhanced surface chemistry from phosphoric acid treatment, which increases surface morphology and porosity for adsorption.

#### 3.4.1 Effect of batch process variables on CIP adsorption

##### 3.4.1.1 Effect of time

**FIGURE 7:**
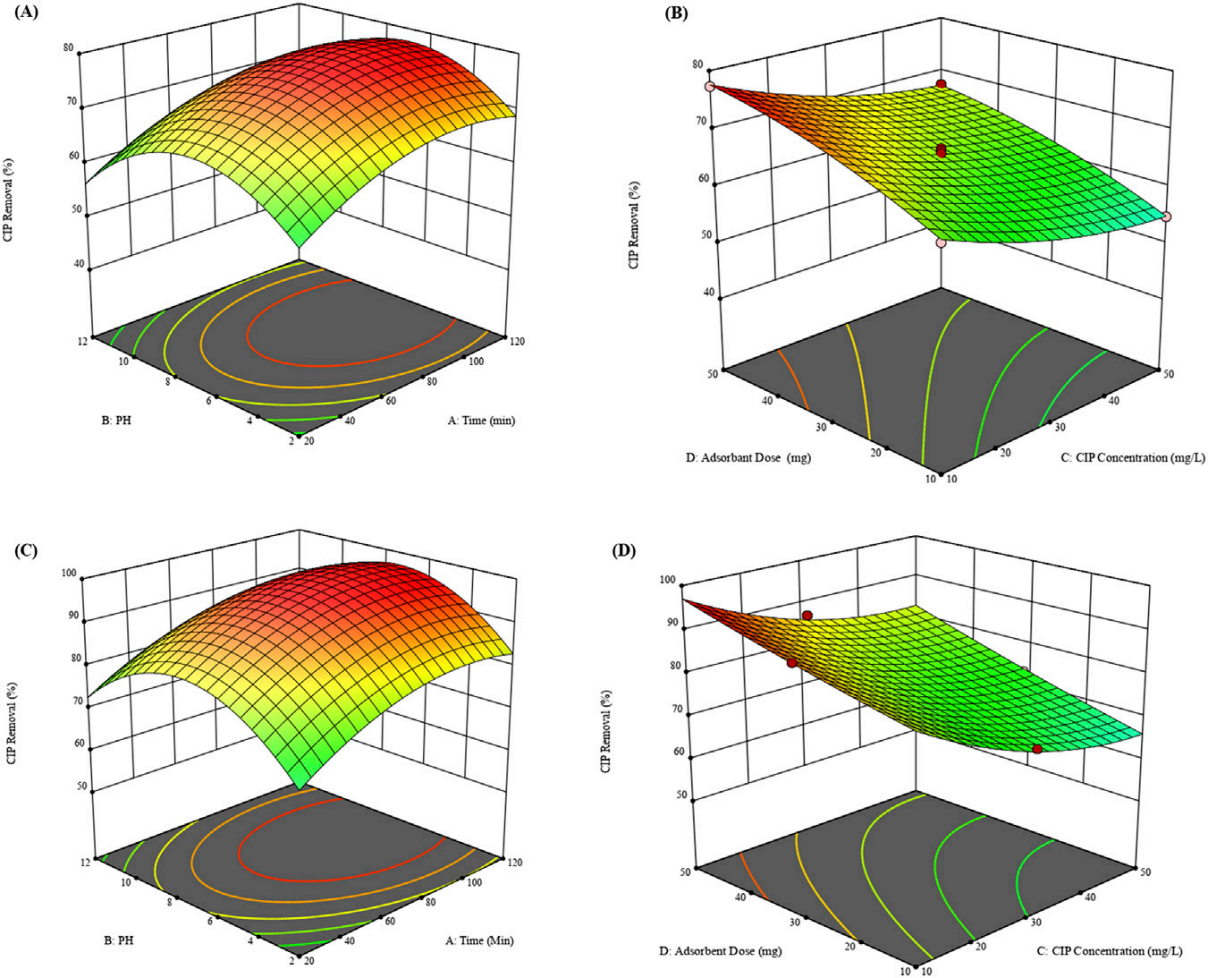
CIP removal (%) response surfaces: CLB10 time vs. pH **(A)** and C_0_ vs. adsorbent dose **(B)**; PAM-CLB1.5-time vs. pH **(C)** and C_0_ vs. adsorbent dose **(D)**.

Contact time generally exhibits a rapid initial increase in adsorbate removal due to the abundance of available active sites and a high concentration gradient. This is followed by a slower phase as the active sites become progressively occupied and intraparticle diffusion within the pores becomes more dominant. Finally, the process reaches equilibrium, where extending the contact time results in little or no additional adsorption (Meseguer et al., 2024). Results of CLB10 in Figure 7A showed significant increases in CIP removal percentage with longer contact time, ranging from 44.59% (at 20 min) to 77.35% (at 70 min). The sharp increase in the CIP removal percentage at initial stages followed by a plateau, reflects that removal is fastest when active sites are unsaturated. As equilibrium is approached at 70 min (Figure 7A), the force for mass transfer decreases due to site saturation and diffusion limitations, which leads to lowering the removal efficiency (De Oliveira et al., 2023). The initial abundance of active sites on the biochar surface facilitates both rapid diffusion and chemical interactions such as hydrogen bonding and π-π electron donor-acceptor mechanisms as contact time proceeds, these processes become less pronounced but still enable additional molecules to reach internal pores and bind, resulting in incremental (though slower) increases in capacity (Zeng et al., 2018). In contrast, PAM-CLB1.5 achieves much higher CIP removal, with a minimum observed value of around 50.54% and a maximum reaching 96.79%, as shown in Figure 7C and Table 9. The higher CIP removal % by PAM-CLB1.5 is due to the improvement in surface structure and surface chemical alteration as supported by the characterization results (SEM and FTIR) in Section 3.1. EDX analysis (Figure 3C also confirms enhanced oxygen and phosphorus signals in PAM-CLB1.5. BET results of PAM-CLB1.5’s (Table 4) also supported its higher surface area, larger pore volume, and greater pore size, enhancing mass transfer and providing more active sites for adsorption (Zhao et al., 2017). Overall, PAM-CLB1.5’s higher CIP removal results from the corresponding effects of improved pore structure and phosphate-rich surface chemistry, as evidenced by Figures 1–3 and Table 4. A comparable “fast-then-plateau” contact-time profile is consistently observed for H_3_PO_4_ modified biochars in CIP adsorption. Consistent with these findings, Iqbal et al. (Iqbal et al., 2022) achieved a 91% CIP removal efficiency using phosphoric-acid modified sidr biochar, with an optimized contact time of roughly 60 min. Similar results were achieved by many other researchers by using modified biochars.

##### 3.4.1.2 Effect of pH

pH is a critical controlling factor in batch adsorption because it governs the ionization/speciation of the contaminant along with the surface charge and functional-group activity of the adsorbent (Hamad, 2026). For the CLB10, CIP adsorption showed a clear U-shaped dependence on pH, with the lowest CIP removal at extreme acidic and basic conditions (pH 2 and 12) as shown in Figure 7A and the highest CIP removal was observed at neutral pH, where CIP mainly exists in its zwitterionic form (Sharma et al., 2025; Wakejo et al., 2022). At low pH, CIP is positively charged, but excessive protonation of the biochar surface (pH_p_zc = 5.94) limits the number of available negatively charged sites and weakens hydrogen bonding, resulting in reduced adsorption. At high pH, both the biochar surface and CIP become negatively charged, causing electrostatic repulsion and a further drop in adsorption efficiency. In contrast, PAM-CLB1.5 follows the same general U-shaped trend but achieves consistently higher removal across the entire pH range, reaching over 95% near neutrality, as shown in Figure 7C. The lower pH_p_zc of PAM-CLB1.5 (3.68) reflects an increased abundance of acidic and phosphate-functional groups, which keep the surface negatively charged across a wider pH range and create more active binding sites (Ma et al., 2020). These modifications strengthen hydrogen bonding and electrostatic interactions (Ouy et al., 2024); allowing PAM-CLB1.5 to sustain high CIP adsorption even when conditions are less favorable, making it more pH-resilient and efficient than CLB10. Our results are consistent with those of Iqbal et al. (Iqbal et al., 2022). Considering CIP speciation, CIP exists mainly in cationic form below pKa_1_ (6.1), predominantly as a zwitterion between pKa_1_ and pKa_2_ (8.7), and mainly as an anion above pKa_2_. Thus, at pH 2, CIP is almost completely cationic, while near the optimized pH values of CLB10 (5.81) and PAM-CLB1.5 (6.60), cationic/zwitterionic species dominate, favoring adsorption through electrostatic attraction, hydrogen bonding, and π–π interactions. At pH 12, CIP becomes predominantly anionic, which reduces adsorption due to electrostatic repulsion with negatively charged biochar surfaces. This explains the higher removal near neutral pH and lower removal under strongly acidic or alkaline conditions.

##### 3.4.1.3 CIP concentration

Increasing the initial CIP concentration typically boosts the mass-transfer driving force and adsorption capacity of biochar, yet it reduces removal efficiency as a finite number of surface sites become saturated, leaving more CIP in solution (Demirdağ et al., 2025). For CLB10, this saturation trend is evident, as shown in Figure 7B, the CIP removal % was dropped from about 78% to 67% with increase in CIP concentration from 10 mg L^-1^–50 mg L^-1^ reflecting sufficient active sites and pores at low CIP concentration which results in more CIP capture via functional group interactions, but at higher CIP concentration there is limited surface area, lower site density, and weaker binding affinity patterns which is consistent with other unmodified agricultural-waste biochars (Tran et al., 2022). In the case of PAM-CLB1.5, as shown in Figure 7D, higher CIP removal % was noted (98% at 10 mg L^-1^ and 83% at 50 mg L^-1^), due to enhanced porosity, more oxygenated/phosphate functional groups, and stronger CIP affinity that delay saturation and provide more high-energy sites (Wang et al., 2025); reflecting findings for H_3_PO_4_-modified biochar which show slower efficiency declines compared to CLB10. Even PAM-CLB1.5’s removal efficiency decreases at the highest concentration, indicating that once its enhanced sites are mostly occupied, available binding spots become limited. This common pattern is seen in various other highly porous biochars and activated carbons used for CIP removal (Hamad et al., 2025).

##### 3.4.1.4 Adsorbent dose

Increasing the adsorbent dose enhance CIP removal efficiency because higher dose means more active surface sites, oxygen-rich functional groups, and pores that can adsorb CIP molecules. This pattern is commonly seen with antibiotics on biochar (Liu and Yang, 2025). For the CLB10, removal efficiency increased from 64% to 78% as the CLB10 dose increases from 10 mg to 50 mg as shown in Figure 7B. Similar results were reported in various studies on agriculturally based biochar (Ripanda et al., 2023). At lower dosages, the CLB10 provides a limited number of active adsorption sites, resulting in competitive interactions among CIP molecules and leaving a substantial fraction unadsorbed once the available sites become saturated (Singh et al., 2022). Higher doses ease that competition by offering more surface area and groups, and results in increase in adsorbate removal rates (Ullah et al., 2025).

The PAM-CLB1.5 showed a much sharper CIP removal from 83% to 97% with increase in dose from 10 mg to 50 mg, as shown in Figure 7. D, aligning with findings that H_3_PO_4_-modified biochars often hit over 95% CIP removal even at low doses due to their high porosity and functional groups (Iqbal et al., 2022). The modification alters the surface area and pore volume (discussed in Section 3.1.4) and adds phosphate groups (like P–O–C and P=O) plus acidic ones (discussed in Section 3.1.2) significantly increasing surface negativity and reactivity to better attract fluoroquinolones like CIP. This facilitates multiple binding mechanisms, including stronger electrostatic attraction with ionized CIP species, enhanced hydrogen bonding via oxygen- and phosphate-containing surface groups, and additional acid–base as well as π–π interactions comparable to those observed in advanced nitrogen-doped or magnetic biochars (Ling et al., 2024).

### 3.5 Process optimization and comparison with other adsorbents

The optimization of CIP adsorption conditions using RSM allowed for an in-depth evaluation of the main operational variables influencing CIP removal efficiency by CLB10 and PAM-CLB1.5 biochars. The optimized process parameters and their corresponding adsorption performance for both studied adsorbents are presented in Table 11. For CLB10 biochar, the RSM model projected a maximum CIP removal efficiency of 77.56%, attained at optimum contact time, initial CIP concentration, and adsorbent dosage of 106.58 min, 5.81 and 48.02 mg, respectively. Validation experiments in triplicate were conducted for CLB10 under these optimized conditions, which produced an observed CIP removal efficiency of 78.43%, closely aligned with the predicted value which confirms the model’s reliability.

**TABLE 11:** Optimization parameters for the CIP removal efficiency for CLB10 and PAM-CLB1.5

Biochar	Time (Min)	PH	CIP concentration (mgL^-1^)	Adsorbent dose (mg)	Predicted CIP removal (%)	Actual CIP removal (%)
CLB10	106.58	5.81	10.27	48.02	77.56	78.43
PAM-CLB1.5	102.50	6.60	11.34	49.03	96.33	97.54

In contrast, the PAM-CLB1.5 exhibited better results, with the RSM predicting a removal efficiency of 96.33% under optimum conditions of contact time, pH, initial CIP concentration and adsorbent dosage of 102.50 min, 6.60, 11.34 mg L^-1^, and 49.03 mg, respectively. Experimental validation under these conditions led to a slightly higher removal efficiency of 97.54%, further verifying the precision of the statistical model. The strong concurrence between predicted and experimental values across both studied biochars in current study reflects the robustness and predictive accuracy of the RSM optimization framework. Moreover, the higher adsorption performance of PAM-CLB1.5 than CLB10 reveals the pivotal role of surface modification in enhancing the affinity of biochar toward CIP molecules. Results in Table 12 shows that both CLB10 and PAM-CLB1.5 have better CIP removal % than many other raw and modified biochars.

**TABLE 12:** Comparison of different adsorbents with CLB10 and PAM-CLB1.5 of the current study for the removal of CIP from water.

S. No.	Adsorbent/Biochar type	Removal (%)	pH	C_0_ (mg L^-1^)	Dose (mg)	Time (min)	References
1	Bamboo sawdust biochar	45	7.5	20	500	46	Sharma et al. (2025)
2	Millet husk biochar	70	8	5	400	1440	Chemtai et al. (2024)
3	KOH-modified Millet husk biochar	88	8	5	400	1440	Chemtai et al. (2024)
4	Fe-modified pine-cone biochar	91.3	6.0	20	100	180	Aziz et al. (2024)
5	Rice straw biochar	20	3	10	750	720	Shao et al. (2024)
6	KMnO_4_-modified Rice-straw biochar	47	3	10	750	720	Shao et al. (2024)
7	KMnO_4_ + NaOH modified Rice-straw biochar	56	3	10	750	720	Shao et al. (2024)
8	Rice-straw biochar	37	7	10	1500	1080	Wang et al. (2025)
9	H_3_PO_4_-modified Rice straw biochar	90	7	10	1500	1080	Wang et al. (2025)
10	Cotton husk biochar	55	6	30	100	360	Du et al. (2025)
11	Mn-modified magnetic dual-sludge biochar	38	5	200	2500	720	Liu and Yang, (2025)
12	CLB10	78.43	5.81	10.27	48.02	106.58	This study
13	PAM-CLB1.5	97.54	6.60	11.34	49.03	102.50	This study

### 3.6 Isotherm

**FIGURE 8:**
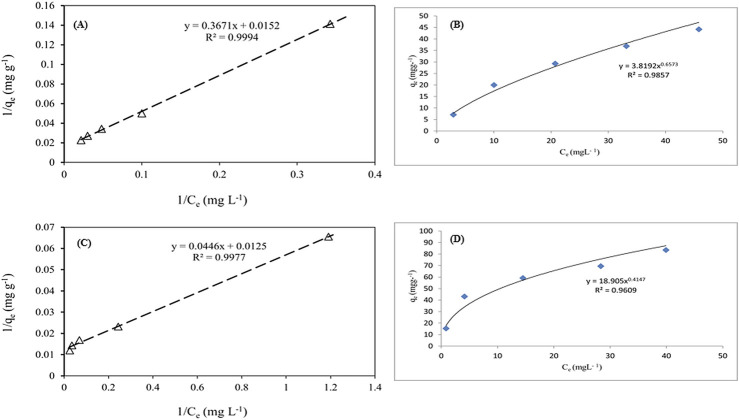
Linearized Langmuir isotherm plots for CIP adsorption using CLB10 **(A)** and PAM-CLB1.5 **(C)**, and non-linear Freundlich isotherm fits for CLB10 **(B)** and PAM-CLB1.5 **(D)**.

The adsorption isotherm analysis for CIP on CLB10 and PAM-CLB1.5 provides critical insights into the nature of the sorption process and surface interactions. The Langmuir model, characterized by a high correlation coefficient (R^2^ = 0.999 for CLB10 and 0.997 for PAM-CLB1.5) as mentioned in Table 12 and Figures 8A,C indicates that CIP adsorption on CLB10 and PAM-CLB1.5 predominantly occurs as a monolayer on a homogeneous surface with finite and energetically equivalent sites based on the model fitting.

Here, the maximum adsorption capacity (Qmax) increased substantially from 65.79 mg/g for the CLB10 to 80 mg/g for PAM-CLB1.5, following phosphoric acid modification, reflecting enhanced surface area and the introduction of functional groups that provide additional binding sites. Similar enhancement in adsorption capacity after phosphoric acid modification has been reported by Iqbal et al. (2022), they found that H_3_PO_4_-modified sidr leaf biochar increased the CIP adsorption capacity from 43.48 to 62.50 mg g^-1^, attributed to the introduction of PO_4_^3-^ groups and improved surface characteristics. The Langmuir constant KL is also increased for PAM-CLB1.5 (0.258 L/g), suggesting stronger affinity between the biochar surface and CIP molecules. The dimensionless separation factor RL further confirms the adsorption favorability, with values closer to zero for PAM-CLB1.5 (RL = 0.037) than CLB10 (RL = 0.194), which indicates more favorable and effective adsorption on the modified biochar (Lu and Zhao, 2024). The Freundlich isotherm model, which supposes heterogeneous adsorption sites and multilayer adsorption, also fits the data well, though with slightly lower R^2^ values of 0.985 for CLB10 and 0.960 for PAM-CLB1.5 as shown in Table 12 and Figures 8B,D. The 1/n values less than one for both studied biochars as shown in Table 12 (CLB10: 0.657, PAM-CLB1.5: 0.414) revealed favorable adsorption intensity and surface heterogeneity, with the modified biochar exhibiting stronger and more heterogeneous binding sites due to phosphoric acid treatment, as reflected in the elevated KF (18.905 for PAM-CLB1.5 vs. 3.819 for CLB10). The adsorption process is more appropriately interpreted as a combined mechanism involving dominant Langmuir-type behavior together with heterogeneous surface interactions. Enhanced micro and mesoporous structures, as well as oxygen and phosphorus containing surface groups (POx) as mentioned in Section 3.1.2 of the current study, support various mechanisms such as π–π interactions, hydrogen bonding, Lewis’s acid–base interactions, and pore-filling, which collectively improve CIP adsorption (Farooq et al., 2024).

These findings align with numerous studies that reported phosphoric acid modification of biochar not only increases surface area and porosity but also introduces rich surface chemistry, boosted adsorption capacities and affinities for pharmaceutical contaminants (Ma et al., 2020).

### 3.7 Kinetics

**FIGURE 9:**
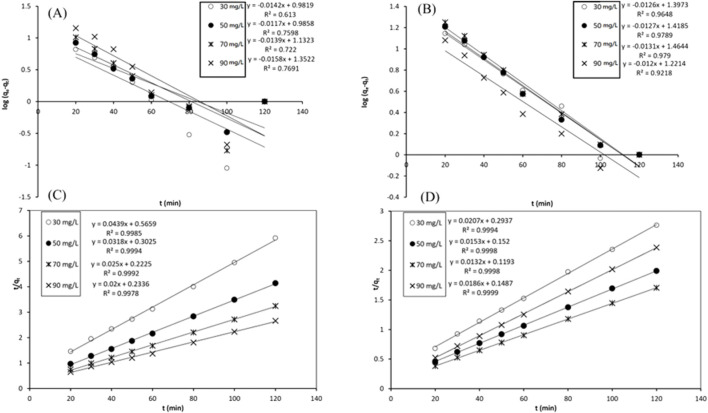
Pseudo-first-order kinetics for CLB10 **(A)** and PAM-CLB1.5 **(B)**, and Pseudo-second-order kinetics for CLB10 **(C)** and PAM-CLB1.5**(D)**.

The kinetic data for CIP adsorption on CLB10 and PAM-CLB1.5 explain key mechanisms and performance differences that are critical for field applications. Results in Figure 9 and Table 13 show that the adsorption data of both CLB10 and PAM-CLB1.5 fitted well into pseudo-second order (PSO) model (0.998 for CLB10 and 0.999 for PAM-CLB1.5), which reflects that adsorption is chemisorption and is controlled by chemical bonding and electron sharing. The calculated adsorption capacities (qe cal) closely match experimental values (Table 13), and the initial adsorption rate (H) is higher for PAM-CLB1.5, demonstrating more rapid adsorption kinetics compared to CLB10. The data in Table 13 indicates that PAM-CLB1.5, achieves significantly higher experimental adsorption capacities (e.g., 43.41–78.31 mg/g) than CLB10 (20.28–44.98 mg/g), which is attributed to the introduction of phosphate groups that enhance surface area, porosity, and the availability of active functional groups which likely contribute to improved adsorption. In contrast, the pseudo-first order (PFO) model, which typically describes physisorption with diffusion-controlled mechanisms, shows weaker correlation and poorer prediction of adsorption capacity, especially for CLB10 (R^2^ as low as 0.613), indicating poorer predictive performance under the investigated conditions. However, pseudo-second-order fitting alone does not conclusively establish chemisorption or identify the sole rate-controlling mechanism. Therefore, adsorption behavior was interpreted collectively using evidence from kinetic modeling, FTIR, EDX, BET, SEM, pH_p_zc, and equilibrium analyses.

**TABLE 13:** Pseudo first and second-order kinetic model parameters for CIP adsorption using CLB10 and PAM-CLB1.5

​	​	Pseudo-second order				Pseudo-first order			
*CIP* concentration (mg L^− 1^)	Adsorbent	q_e exp_ (mg g^− 1)^	q_e__cal_ (mg g^− 1^)	K_2_ (g mg^− 1^ min^− 1^)	R^2^	H (mg g^−1^ min^− 1^)	q_e__cal_ (mg g^− 1^)	K_1_ (min^− 1^)	R^2^
*30*	CLB10	20.28	22.779	0.003	0.998	1.767	9.591	−0.032	0.613
	PAM-CLB1.5	43.41	48.309	0.001	0.999	3.404	24.963	−0.029	0.964
*50*	CLB10	28.98	31.446	0.003	0.999	3.305	9.678	−0.026	0.759
	PAM-CLB1.5	60.28	65.359	0.001	0.999	6.578	26.211	−0.029	0.978
*70*	CLB10	36.94	40	0.002	0.999	4.494	13.561	−0.032	0.722
	PAM-CLB1.5	70.35	75.757	0.001	0.999	8.382	29.133	−0.030	0.979
*90*	CLB10	44.98	50	0.001	0.997	4.280	13.652	−0.036	0.769
	PAM-CLB1.5	78.31	81.300	0.002	0.999	17.006	17.131	−0.027	0.918

### 3.8 Regeneration

From a sustainability perspective, the regeneration and reuse of adsorbents are as crucial as their initial adsorption capacity. For biochar and related carbon materials, reactivation after use enhances lifespan, reduces solid waste, and minimizes disposal needs. In antibiotic removal, such as CIP, biomass-based adsorbents typically retain 70%–85% of their initial capacity after four to five regeneration cycles, demonstrating their promise for large-scale and long-term wastewater applications (Liu and Yang, 2025).

**FIGURE 10:**
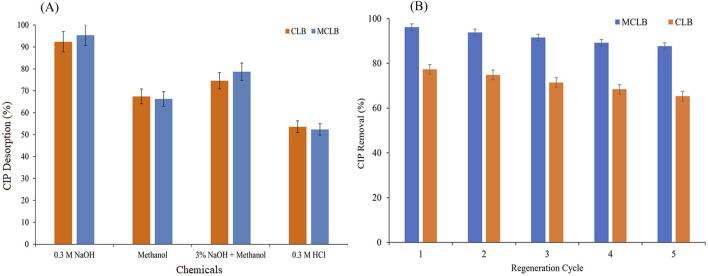
Comparative regeneration efficiency of CLB10 and MCLB **(A)** Effect of different chemical reagents on CIP desorption and **(B)** CIP removal efficiency over five regeneration cycles using 0.3 M NaOH.

A regeneration study was conducted to evaluate the feasibility of CLB10 and PAM-CLB1.5 in the CIP adsorption. Various CIP desorption solutions such as methanol, dilute HCl, 3% NaOH + methanol, and NaOH have been used in previous studies and thus, selected and tested in CLB10 and PAM-CLB1.5 regeneration study. The CIP removal efficiency of CLB10 after first cycle desorption with 0.3 M NaOH, methanol, 3% NaOH + methanol, and 0.3M HCl was 92.34, 67.43, 74.55, and 53.59%, respectively and for PAM-CLB1.5 it was 95.34, 66.28, 78.71, and 52.36% as shown in Figure 10A. Hence, 0.3 M NaOH was used as an eluent for CIP desorption throughout the regeneration study. A 0.3 M NaOH solution is often the most effective solvent for CIP desorption because it strongly promotes electrostatic repulsion and disrupts surface interactions (Wakejo et al., 2022). Under alkaline conditions, CIP becomes mainly anionic (pKa_1_ = 6.1, pKa_2_ = 8.7), while the biochar surface becomes increasingly deprotonated and negatively charged. This dual effect weakens the electrostatic and hydrogen bonding forces responsible for CIP adsorption. The high OH^−^ concentration also competes for active sites, further aiding desorption. In contrast, methanol is less effective since CIP adsorption depends primarily on pH-sensitive electrostatic and complexation processes, not hydrophobic interactions (Alsawy et al., 2022). Similarly, HCl decreases desorption by protonating CIP and surface groups, increasing attraction rather than promoting release.

After five sequential adsorption/desorption cycles, the removal of CIP by CLB10 and PAM-CLB1.5 varied from 77.35 to 65.38 (11.97% reduction) and 96.14 to 87.66 (reduction of 8.48%) as shown in Figure 10B. The reusability studies revealed that 87% of CIP can be removed even in the fifth cycle, suggesting enhanced stability and reusability of PAM-CLB1.5. The reusability of PAM-CLB1.5 without significant loss in CIP removal is one of the benefits of this process regarding environmental concerns and economic feasibility. Although the batch adsorption and regeneration results demonstrate promising CIP removal and adsorbent stability, practical water systems contain dissolved salts, natural organic matter, and competing ions that may alter adsorption behavior. Therefore, evaluation under multi-component systems, real wastewater conditions, and continuous-flow operation is required for comprehensive applicability assessment.

### 3.9 Practical feasibility, cost, and environmental considerations

Phosphoric-acid modification significantly improved CIP adsorption; however, practical implementation requires consideration of production cost, scalability, and environmental impacts. In the present study, the estimated production cost per kg of CLB10 and PAM-CLB1.5 was approximately 3.52 $ and 27.47 $, respectively (Supplementary Tables A3, A4), reflecting contributions from pyrolysis, chemical modification, and post-treatment operations. The use of cauliflower-leaf agricultural waste as feedstock supports cost reduction and biomass valorization.

Despite its effectiveness, H_3_PO_4_ modification involves additional chemical consumption, washing, and potential generation of acidic effluents requiring appropriate management. Nevertheless, phosphoric acid remains a widely used activating agent because of its ability to promote pore development and surface functionalization under moderate processing conditions (Sizmur et al., 2017). The regeneration performance observed in this study further supports the feasibility of the modified adsorbent by potentially reducing replacement frequency and operating costs. However, comprehensive techno-economic assessment, environmental life-cycle analysis, and scale-up validation remain necessary for industrial application (Tan et al., 2015).

## 4 Conclusion

In this study, cauliflower leaves biochar in both raw (CLB10) and modified (PAM-CLB1.5) form was used for CIP removal from water. Process optimization of CLB10 was done using Box–Behnken response surface methodology (RSM) at both the biochar preparation stage and in batch adsorption. At the biochar preparation stage, the effects of biochar preparation conditions, i.e., pyrolysis time, pyrolysis temperature, and biomass particle size on CIP adsorption were studied, and the optimum conditions were observed at a time, temperature, and particle size of 90 min, 475 °C, and 125 μm, respectively. CLB10 achieved maximum CIP removal of 78.43% at pH, adsorbent dose, initial CIP concentration, and contact time of 5.81, 48.02 mg, 10.27 mg L^-1^, and 106.58 min, respectively. On the other hand, PAM-CLB1.5 achieved a higher CIP removal of 97.54% at pH, adsorbent dose, initial CIP concentration, and contact time of 6.60, 49.03 mg, 11.34 mg L^-1^, and 102.50 min, respectively. PAM-CLB1.5 showed better results than CLB10 as phosphorous acid modification increased its texture, surface functional groups, surface area, pore volume, and average pore size, as supported through BET, FTIR, SEM and EDX analyses. Isotherm analysis showed that the Langmuir model best described CIP uptake with monolayer capacities of 65.79 and 80.00 mg g^-1^ for CLB10 and PAM-CLB1.5, respectively, indicating adsorption mainly on homogeneous sites. Kinetic data for both adsorbents followed the pseudo-second-order model with very high correlation coefficients (R^2^ ≥ 0.997 for CLB10 and ≥0.999 for PAM-CLB1.5), pointing to chemisorption-controlled uptake for CIP removal. Therefore, the superior CIP removal by PAM-CLB1.5 is attributed to synergistic effects of improved pore accessibility, increased pore volume, phosphate/oxygen-rich functional groups, reduced pH_p_zc, and stronger electrostatic, hydrogen-bonding, and π–π interactions, rather than BET surface area alone. It was concluded that PAM-CLB1.5 can be used as potential alternatives to expensive commercial adsorbents for CIP removal from water. However, it is recommended that further studies should be carried out on thermodynamics, biochar yield, C-H-N-O analysis, advanced models such as Sips, Redlich–Peterson, and intra-particle diffusion analysis and on influence of competing ions to evaluate the adsorbent performance under continuous-flow and real wastewater conditions to further assess its practical applicability at larger scales.

## Data Availability

The original contributions presented in the study are included in the article/Supplementary Material, further inquiries can be directed to the corresponding author.
