# Supplementary material for: Enhanced adsorption of ciprofloxacin from water using raw and phosphoric acid-modified cauliflower-leaves derived biochar: response surface optimization
Source: Front Chem. 2026 Jul 23;14:1852792. doi: 10.3389/fchem.2026.1852792 (PMC13442430; doi:10.3389/fchem.2026.1852792)
Supplement: Supplementary file 1 [file Table1.docx]

Supplementary Table 1

**Table A1. List of biochar prepared with removal efficiency for CIP**

| **S. No** | **Biochar** | **CIP removal (%)** |
| --- | --- | --- |
| 1. | CLB1 | 57.58 |
| 2. | CLB2 | 65.82 |
| 3. | CLB3 | 53.34 |
| 4. | CLB4 | 61.42 |
| 5. | CLB5 | 66.23 |
| 6. | CLB6 | 57.34 |
| 7. | CLB7 | 55.26 |
| 8. | CLB8 | 66.98 |
| 9. | CLB9 | 57.18 |
| 10. | CLB10 | 67.06 |
| 11. | CLB11 | 52.87 |
| 12. | CLB12 | 59.38 |
| 13. | CLB13 | 66.05 |
| 14. | CLB14 | 61.42 |
| 15. | CLB15 | 47.78 |
| 16. | CLB16 | 62.15 |
| 17. | CLB17 | 63.88 |
