# Supplementary material for: Enhanced adsorption of ciprofloxacin from water using raw and phosphoric acid-modified cauliflower-leaves derived biochar: response surface optimization
Source: Front Chem. 2026 Jul 23;14:1852792. doi: 10.3389/fchem.2026.1852792 (PMC13442430; doi:10.3389/fchem.2026.1852792)
Supplement: Supplementary file 2 [file Table2.docx]

Supplementary Table 2

**Table A2. List of modified biochar with removal efficiency for CIP**

| **S. No** | **Modified Biochar** | **CIP removal (%)** |
| --- | --- | --- |
| 1. | NAM-CLB0.5 | 79.04 |
| 2. | NAM-CLB1.5 | 80.07 |
| 3. | NAM-CLB2.5 | 76.95 |
| 4. | PAM-CLB0.5 | 79.83 |
| 5. | PAM-CLB1.5 | 82.77 |
| 6. | PAM-CLB2.5 | 73.53 |
| 7. | AAM-CLB0.5 | 78.18 |
| 8. | AAM-CLB1.5 | 79.03 |
| 9. | AAM-CLB2.5 | 75.03 |
| 10. | PHM-CLB0.5 | 77.99 |
| 11. | PHM-CLB1.5 | 80.97 |
| 12. | PHM-CLB2.5 | 78.07 |
| 13. | AHM-CLB0.5 | 72.97 |
| 14. | AHM-CLB1.5 | 73.42 |
| 15. | AHM-CLB2.5 | 71.44 |
| 16. | SBM-CLB0.5 | 73.88 |
| 17. | SBM-CLB1.5 | 72.93 |
| 18. | SBM-CLB2.5 | 69.07 |
| 19. | ZCM-CLB0.5 | 71.77 |
| 20. | ZCM-CLB1.5 | 73.72 |
| 21. | ZCM-CLB2.5 | 72.57 |
| 22. | CCM-CLB0.5 | 71.76 |
| 23. | CCM-CLB1.5 | 72.77 |
| 24. | CCM-CLB2.5 | 70.47 |
| 25. | KCM-CLB0.5 | 74.67 |
| 26. | KCM-CLB1.5 | 75.37 |
| 27. | KCM-CLB2.5 | 72.27 |
