# Supplementary material for: Enhanced adsorption of ciprofloxacin from water using raw and phosphoric acid-modified cauliflower-leaves derived biochar: response surface optimization
Source: Front Chem. 2026 Jul 23;14:1852792. doi: 10.3389/fchem.2026.1852792 (PMC13442430; doi:10.3389/fchem.2026.1852792)
Supplement: Supplementary file 3 [file Table3.docx]

Supplementary Table 3

**Table A3. Cost analysis for the preparation of 1 kg CLB10**

| **S. No** | **Cost Component** | **Unit** | **Quantity Required for 1 kg Biochar** | **Unit Cost (PKR)** | **Total Cost (PKR)** |
| --- | --- | --- | --- | --- | --- |
| 1 | Cauliflower leaf biomass | Kg | 2.39 | 1 | 2.39 |
| 2 | Washing water | L | 5 | 2 | 10 |
| 3 | Oven drying electricity (60°C, 12 h) | kwh | 4 | 45.70 | 182.70 |
| 4 | Grinding/milling cost | kg | 5 | 2.5 | 12.5 |
| 5 | Sieving cost | kg | 2 | 2.5 | 5 |
| 6 | Nitrogen gas (100 mL min⁻¹ during pyrolysis | L | 9 | 70 | 630 |
| 7 | Furnace electricity (300–650°C pyrolysis) | kwh | 3 | 45.70 | 137.1 |
| Total |  |  |  |  | 979.69 Pkr/3.52 USD |
