# Supplementary material for: Enhanced adsorption of ciprofloxacin from water using raw and phosphoric acid-modified cauliflower-leaves derived biochar: response surface optimization
Source: Front Chem. 2026 Jul 23;14:1852792. doi: 10.3389/fchem.2026.1852792 (PMC13442430; doi:10.3389/fchem.2026.1852792)
Supplement: Supplementary file 4 [file Table4.docx]

Supplementary Table 4

**Table A4. Cost analysis for preparation of 1 kg PAM-CLB1.5**

| **S. No** | **Cost Component** | **Unit** | **Quantity Required for 1 kg Modified Biochar** | **Unit Cost (PKR)** | **Total Cost (PKR)** |
| --- | --- | --- | --- | --- | --- |
| 1 | Raw biochar | kg | 1 | 979.69 | 979.69 |
| 2 | H₃PO₄ | L | 4.90 | 1350 | 6615 |
| 3 | Deionized water | L | 33.33 | 2 | 66.66 |
| 4 | Shaking/incubation electricity (24 h, 220 rpm) | kwh | 2 | 45.70 | 91.4 |
| 5 | Washing water after treatment | L | 10 | 2 | 20 |
| 6 | Oven drying electricity | kwh | 4 | 45.70 | 182.70 |
| Total |  |  |  |  | 7652PKR/27.47 USD |
